# Supplementary material for: Preparing high-concentration individualized carbon nanotubes for industrial separation of multiple single-chirality species
Source: Nat Commun. 2023 Apr 29;14:2491. doi: 10.1038/s41467-023-38133-0 (PMC10148823; doi:10.1038/s41467-023-38133-0)
Supplement: Supplementary file 1 — Supplementary Information [file 41467_2023_38133_MOESM1_ESM.pdf]

Supplementary Information

Preparing High-Concentration Individualized Carbon Nanotubes for Industrial Separation of Multiple Single-Chirality Species

Dehua Yang, [1,2,3] Linhai Li, [1,2,4] Xiao Li, [1,2,4] Wei Xi, [1,2] Yuejuan Zhang, [1,2,4] Yumin Liu,[1] Xiaojun Wei, [1,2,4,5] Weiya Zhou, [1,2,4,5] Fei Wei,[6] Sishen Xie, [1,2,4,5] and Huaping Liu[1,2,4,5,*]

[1]Beijing National Laboratory for Condensed Matter Physics, Institute of Physics, Chinese Academy of Sciences, Beijing 100190, China

[2]Center of Materials Science and Optoelectronics Engineering, and school of Physical Sciences, University of Chinese Academy of Sciences, Beijing 100049, China

[3]Advanced Passivation Technology Lab, College of Physics Science and Technology, Hebei University, Baoding 071002, China

[4]Beijing Key Laboratory for Advanced Functional Materials and Structure Research, Beijing 100190, China

[5]Songshan Lake Materials Laboratory, Dongguan, Guangdong 523808, China

[6]Department of Chemical Engineering, Tsinghua University, Beijing 10084, China

Corresponding author: liuhuaping@iphy.ac.cn

Supplementary Note 1: Experimental details

1.1 Preparation of high-concentration individualized single-wall carbon nanotube (SWCNT) solution:

The SWCNT solution with initial concentration of 1 mg/mL were prepared by traditional method. Specifically, 100 mg of raw materials (HiPco, Fluffy Powder, batch no. HR32-152, NanoIntegris, Inc.) was dispersed in 100 mL of aqueous solution of sodium dodecyl sulfate (SDS, 99%, Sigma-Aldrich) with a concentration of 2 wt%. Ultrasonic dispersion was performed for 9 hours at an ultrasonic power density of 0.3 W/mL by tip-type homogenizer (Sonifier 450D, Branson) at 15 °C. Subsequently, insoluble impurities and large bundles were removed by centrifugation for 1 hour at the centrifuge force of 210,000 g (S50A, Hitachi CS150FNX). Then, 90% of the supernatant was collected as the SWCNT dispersions.

High-concentration SWCNT solution are prepared by redispersion method, in which SWCNT solution is firstly ultrasonically dispersed, followed by centrifugation and re-ultrasonic dispersion (as shown in Fig. 1a in the main text). For 100-mL SWCNT dispersion, for every 1-mg/mL increase in concentration, the time of the first and second ultrasonication was increased by 5 hours and 20 minutes, respectively. Taking the dispersion of a SWCNT solution at an initial concentration of 4 mg/mL as an example, 400 mg of raw HiPco-SWCNTs was added to 100 mL of aqueous solution of 2 wt% SDS. Ultrasonic dispersion was performed for 24 hours at an ultrasonic power density of 0.3 W/mL at 15 °C. Subsequently, insoluble impurities and large bundles were removed by centrifugation for 1 hour at 210,000 g (S50A, Hitachi CS150FNX). And 95% of the supernatant was collected and ultrasonically dispersed for another 1 hour. After that, the second centrifugation was performed for 15 min at the centrifuge force of 210,000 g to remove the remained carbon impurities and the metallic particles generated from the ultrasonic tips. 95% of the supernatant was collected as the as-prepared mono-dispersion for subsequent separation of single-chirality species. The dispersion of high-concentration G-SWCNTs (GNH-1200 Beijing North Guonemg Technology Co., Ltd) and Tuball SWCNT (carbon ≥ 80%, 1.2-2.0 nm in diameter,

Sigma-Aldrich) was performed by the same process as HiPco-SWCNTs.

1.2 Preparation of high-concentration SWCNT solution by traditional methods:

Compared with the redispersion method, the traditional dispersion method only has one ultrasonic dispersion and one centrifugal process. The dispersion time is dependent on the SWCNT concentration. Taking the dispersion of a SWCNT solution at an initial concentration of 4 mg/mL as an example, 400 mg of HiPco-SWCNT powder was dispersed into 100-mL aqueous solution of 2 wt% SDS at a power density of 0.3 W/mL. To sufficiently disperse SWCNTs, the dispersion time was varied from 16 to 32 hours to investigate the dispersity of SWCNTs. The as-dispersed SWCNT solution was centrifuged at a centrifugal force of 210000×g to remove the undispersed SWCNT bundles and impurities. To obtain individualized SWCNT solution, the centrifugation time was increased from 1 to 2 hours. After centrifugation, 90% of the supernatant was collected as parent solution for single-chirality separation.

1.3 Evaluation of the dispersibility of SWCNT solution

The dispersity of the as-prepared SWCNT solution is evaluated by the separation of (6, 4) single-chirality SWCNTs. Specifically, 10 mL of SWCNT dispersion was loaded into a 40-mL gel column at 10 °C. The (6, 4) SWCNTs was selectively adsorbed in the gel column and eluted by aqueous solution of 5 wt % SDS. The structure distribution of the selectively adsorbed on the gel column was characterized by optical absorption spectra. Comparison of the dispersity of high-concentration SWCNT solution prepared by conventional and current redispersion methods are shown in supplementary Note 3 and supplementary Fig. 3.

1.4 Separation of single-chirality (n, m) species

To investigate the effect of SWCNT concentration on the yield of single-chirality species, 10 mL of the as-prepared SWCNT dispersion with different concentrations was loaded into a gel column of 40 mL equilibrated by 2 wt% SDS at 18 °C. The unadsorbed SWCNTs were eluted by 2 wt% SDS. SC was stepwise introduced to replace the SDS surfactant in column to binary surfactants of 0.5 wt% SC/0.5 wt% SDS. After the replacement of surfactant, the separation temperature was increased to 25 °C. Then, the

adsorbed SWCNTs were selectively eluted with the co-surfactants of X wt % DOC/0.5 wt % SC/0.5 wt % SDS, where the concentration of DOC was increased in a stepwise manner from 0.02 wt% to 0.08 wt% at a step of 0.01 wt % until targeted SWCNTs were eluted. The volume of each eluent is 1.5 CV (column volume). Then, the unadsorbed solution was diluted to 1.5 wt% SDS and successively loaded to the column at 14 °C, 18 °C and 22 °C followed by the same procedure of stepwise elution as described above. In this manner, the separation of multiple single-chirality species with large chiral angles such as (6, 4), (6, 5), (8, 4), (7, 5) and (7, 6) were achieved. The separation of SWCNTs with small chiral angles such as (7, 3), (8, 3), (9, 1), (9, 4), (10, 3) was achieved by the selective adsorption at 22 °C owing to removal of SWCNTs with large chiral angles in the former steps. Subsequently, the SWCNTs with larger diameters and smaller chiral angles such as (12, 1), (11, 1), (10, 2) and (11, 0) were extracted from the final unadsorbed solution by selectively adsorbing into gel in the binary surfactants of 1 wt% SDS/ 0.5 wt% SC system at 16°C. The SWCNTs adsorbed in gel columns were eluted stepwise by gradually increasing the concentration of DOC in the co-surfactants of SDS/SC/DOC, so as to achieve the separation of single-chirality species. The separation temperature was controlled by keeping the whole system including gel columns, SWCNT dispersions and surfactant solutions in a homemade thermostat. The separation procedure was also illustrated in supplementary Fig. 4.

1.5 Mass separation of single-chirality species

As shown in Fig. 1d in the main text, the initial concentration of 4 mg/mL could be optimal concentration from the perspective of the full utilization of raw materials and subsequent separation efficiency. For this, the individualized SWCNT solution with an initial concentration of 4 mg/mL were used as parent solution for mass separation of single-chirality SWCNTs. A XK 50/60 (GE Healthcare) gel column filled with 900 mL of Sephacryl S-200 gel was connected to an automatic chromatography system (Avant 150 GE Healthcare). The as-dispersed individualized SWCNT solution of 360 mL was loaded into the gel column for single-chirality separation using the same procedure as mentioned in supplementary Note 1.4. In this way, milligram-scale

separation of single-chirality species was achieved. The results are shown in Fig. 2 in the main text.

Mass separation of single-chirality species from G-SWCNTs was performed by similar procedure. Considering the difference in content of each (*n, m*) species between G-SWCNTs and HiPco-SWCNTs, 1600 mL of SDS-dispersed G-SWCNTs with the initial concentration of 4 mg/mL was loaded to a XK50/60 column filled with 900 mL of Sephacryl S-200 gel. The separation procedure mentioned in section 4 was followed. In this way, sub-milligram separation of single-chirality species such as (6, 4), (6, 5) (7, 3), (7, 5), (7, 6), (8, 4), (9, 1), (9, 4) and (10, 3) was achieved. The separation temperature was controlled by keeping the whole system, including columns, SWCNT dispersions and surfactant solutions in a homemade thermostat.

1.6 Characterization

Shear viscosity of SWCNT dispersions was measured using a rotational viscometer (NDJ-5S, Qingdao Juchuang Co., Ltd) equipped with a L0 rotor for low-viscosity measurement. Since the dispersions of SWCNTs behave in Newtonian manner for the tested concentrations[1], the shear rate is fixed at 60 rpm. Notably, although the sonication was performed in a water bath fixed at 15°C, the measured temperature of SWCNT dispersions was stabilized at approximately 35°C during sonication due to heat generated by the tip of homogenizer. Therefore, the viscosity of SWCNT dispersions was measured at 35°C.

Optical adsorption spectra of SWCNTs were recorded from 220 to 1350 nm using an ultraviolet-near-infrared spectrophotometer (UV-3600, Shimadzu). The optical path length of quartz cuvette is 10 mm. Raman spectra of SWCNTs were measured using a confocal Raman microscope (HR800, Horiba) coupled with a charge-coupled device detector. The morphology and structure of SWCNTs was characterized by a field emission scanning electron microscope (S-4800, Hitachi), a transmission electron microscope (JEM-2100Plus, JEOL) and an atomic force microscope (MultiMode 8, Bruker).

1.7. Life cycle assessment methodology

Goal and Scope Definition: The goal of this life cycle assessment (LCA) is employed to evaluate the environmental impacts, energy demand and techno-electronic cost of our current separation technique. The scope is limited in the synthesis of raw SWCNTs and subsequent separation of single-chirality SWCNTs. The results of LCA can provide a reference for the development of efficient, environmentally friendly and low-cost separation strategy, so as to promote the industrial production and commercial application of single-chirality SWCNTs.

Life Cycle Inventory (LCI): The separation process was modeled according to the actual parameters of separating single-chirality SWCNTs. The materials and energy input, waste output, and the yields of single-chirality SWCNTs were measured during experiments and calculated accordingly. The volumes of flow-through solutions were measured by the automatic chromatography system.

Life cycle impact assessment: The LCI was analyzed using SimaPro v9.4 software with Ecoinvent 3.8 database[2]. The LCI and Intergovernmental panel on climate change (IPCC) method were used for modeling life cycle impact assessment (LCIA). The life cycle greenhouse gas emissions were calculated based on the global warming potential (GWP) with a timeframe of 100 years contained in IPCC method.

Supplementary Note 2: Effect of sonication time on the preparation of high-concentration SWCNT solution.

During dispersion, scaling sonication duration with SWCNT concentration is very important. To confirm this, we have done the following experiments. 20-mL dispersions with initial concentrations of 1, 4 and 8 mg/mL were prepared by sonicating for identical 2 hours and subsequently centrifugating for 1 hour at 210,000 g. For comparison, a control group of samples with initial concentrations of 4 and 8 mg/mL were sonicated for 6 and 11 hours, respectively. Redispersion was not performed for these two groups. After the identical sonication and ultracentrifugation, the concentrations of the as-prepared dispersions increase with an increase in initial SWCNT concentrations, as shown in supplementary Fig. 1. More importantly, for SWCNT solutions with initial concentrations of 4 and 8 mg /mL, the concentrations of the resulted dispersion solution increase significantly with the extension of ultrasonic time to 4 and 9 h, respectively. Therefore, scaling sonication duration with SWCNT concentration is very important to fully disperse them to increase the concentration of the individualized SWCNT solution and thus improve their separation efficiency.

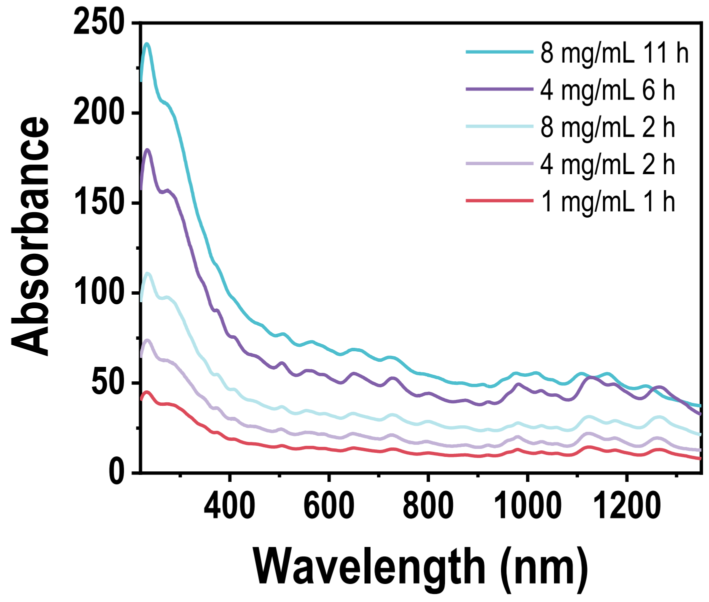

Supplementary Figure 1. Optical absorption spectra of dispersions with different initial concentrations subjected to different sonication time.

To understand suspension evolution and dynamics during tip horn sonication, we

explored the viscosity variation of SWCNT dispersions with initial concentrations of 1 and 4 mg/mL over time. As shown in supplementary Fig. 2a, the viscosity of two dispersions exhibits similar trend over time. At the beginning, the viscosity increases rapidly and then reach the maximum values with increasing sonication duration. With continuously increasing sonication time, the viscosity of SWCNT solutions decreases dramatically and finally reaches an approximate constant value.

Based on the viscosity variation of SWCNTs over sonication time, we proposed the dispersion process of SWCNTs over time. At the initial stage, SWCNT powders floated on the surface of the solution or form precipitate at the bottom. The viscosity is merely derived from the water and surfactants. Subsequently, SWCNT powders were crushed into particles composed of large bundles and impurities. Due to the low dispersity and increased number of suspended particles, the friction between these particles increases the viscosity of solutions. As the sonication time increased, SWCNTs were continuously stripped from the bundles. The sidewalls of SWCNTs exposed to the solution were readily coated with surfactant molecules, decreasing the friction due to high lubrication effect of surfactants[3]. Meanwhile, denser surfactant coatings around SWCNTs provided a repulsive region and thus excellent fluidity[4].

During the dispersion process, the viscosity of the SWCNT solution with an initial concentration of 4 mg/mL was much higher than that of the SWCNT solution with a concentration of 1 mg/mL, especially for the maximum viscosity. Moreover, the viscosity growth stage lasted for 40 minutes, which is also much longer. These results indicate that viscosity has a significant impact on the dispersion of SWCNTs for higher initial concentration. As shown in supplementary Figs. 2b-d, the concentration of the remained SWCNTs in the supernatant decrease rapidly with a decrease in sonication time, indicating that most of the SWCNTs were still present in bundles in a short sonication time and precipitated during centrifugation. These results further confirmed that scaling sonication duration with SWCNT concentration is very important to fully disperse SWCNTs. As shown in Fig. 1b in the main manuscript, the resulting viscosity differed slightly over the wide concentration range of 1-8 mg/mL after first

centrifugation. Besides, we observed little variation in SWCNT concentration and viscosity after the second centrifugation, which evidenced the relatively high efficiency dispersion during the second sonication due to the removal of large bundles and impurities via the first centrifugation.

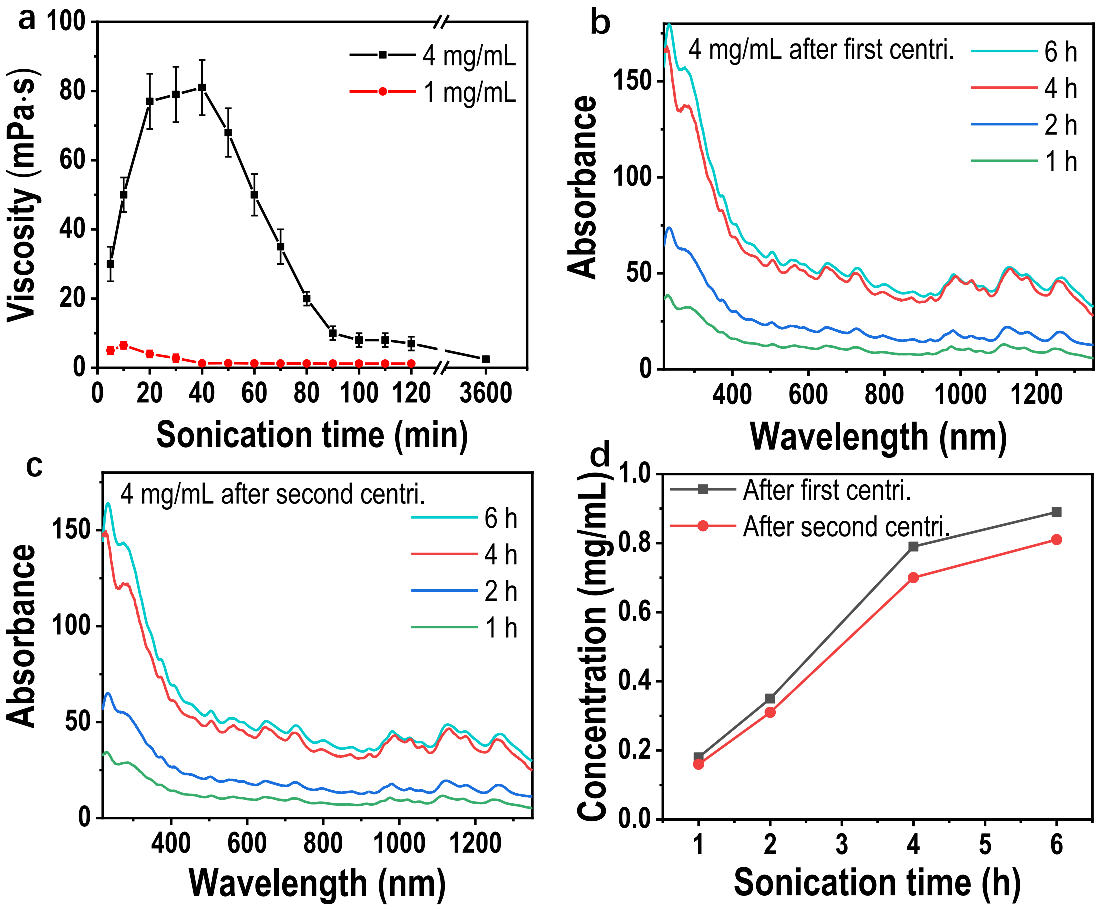

Supplementary Figure 2. Time-dependent variation of solution viscosity and corresponding SWCNT concentration following centrifugation. a) Relationship between sonication time and viscosity of SWCNT solutions with the initial concentration of 1 mg/mL and 4 mg/mL. b)-c) Optical adsorption spectra of SWCNT solutions after first b) and second c) ultracentrifugation. The durations of the first sonication period were shown in the legend. The time of the second sonication was fixed at 15 minutes for all samples. d) Variation of the SWCNT concentration after centrifugation with respect to duration of the first sonication period. "Centri" is the abbreviation of the word "centrifugation".

Supplementary Note 3: Comparison of the dispersity of high-concentration SWCNT solution prepared by conventional and current redispersion methods.

As shown in supplementary Fig. 3a, the separation of (6, 4) single-chirality species cannot be achieved from the SWCNT solution with an initial concentration of 4 mg/mL dispersed by traditional method even if the dispersion time was increased from 16 hours to 32 hours followed by centrifugation for 1 hour, indicating that the as-prepared SWCNT solution was not highly dispersed. The reason why single-chirality (6, 4) SWCNTs cannot be obtained is that the dispersion solution is not sufficiently dispersed. (6, 4) SWCNTs are still mixed with other (*n, m*) SWCNTs in the form of bundles. After loading into gel column, (6, 4) nanotubes that bundled with other (*n, m*) species were adsorbed onto the gel, leading to a low purity of separated (6, 4). Therefore, the separation of (6,4) SWCNTs can be used to evaluate the SWCNT dispersity. Given that the structure distribution of the separated SWCNTs decrease with increasing sonication time, as shown in supplementary Fig. 3a, we assumed that the size of bundles remained in the solutions decreased. And, it is difficult to remove these small bundles by 1-h ultracentrifugation. To obtain the individualized SWCNT solution, the alternative method is to increase the centrifugation time. For HiPco-SWCNT solution with an initial concentration of 4 mg/mL, the centrifugation time increased from 1 to 2 hours after dispersing for 32 hours. After a 2-hour ultracentrifugation, the separation purity distinctly increased accompanied by a decrease in the SWCNT concentration (as shown in supplementary Fig. 3b**)**. Compared with the individualized SWCNT solution with an initial concentration of 1 mg/mL, the SWCNT concentration did not increase clearly, resulting in the low yield of (6, 4) SWCNTs (Supplementary Fig. 3c). To further confirm whether bundles were present in the solutions with initial concentration of 4 mg/mL prepared by the traditional method, the SWCNTs with and without a redispersion were deposited on a Si/SiO_2 and characterized by AFM. More bundles were observed in the AFM image of SWCNTs prepared by sonication for 24 h followed by ultracentrifugation for 1 h (Supplementary Fig. 3d). In contrast, Supplementary Figure 3e shows the well dispersed SWCNTs which were prepared by the redispersion

method, in which the SWCNT samples were redispersed for another 1 h and centrifugated for 15 min. These results fully indicate that it is not possible to prepare high-concentration and high-dispersity SWCNT solution using the traditional preparation method.

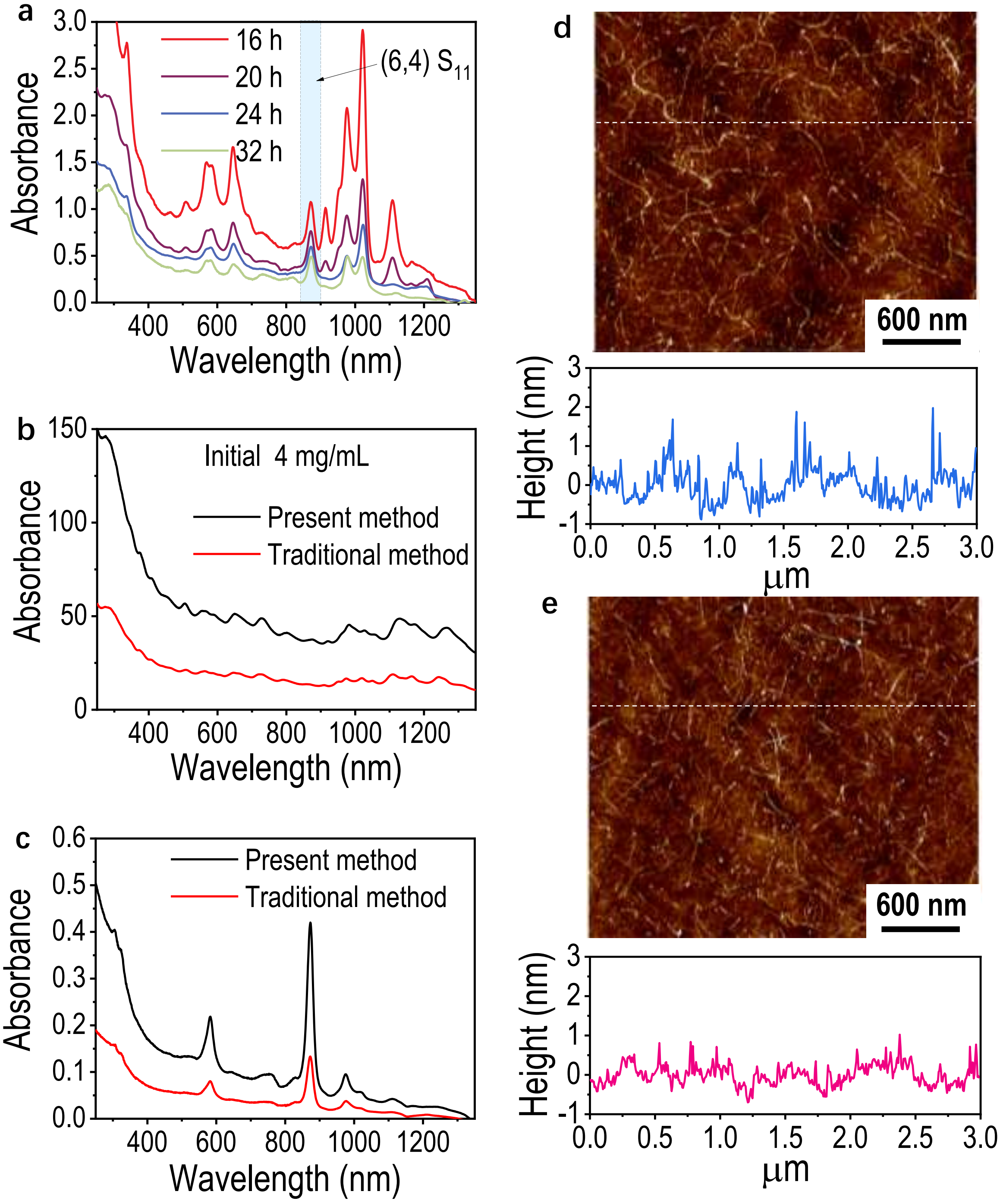

Supplementary Figure 3. Comparison of the dispersity of high-concentration SWCNT solution prepared by conventional and current redispersion methods. (a) Optical adsorption spectra of the SWCNTs separated from high-concentration SWCNT

solutions prepared by conventional methods. The eluted SWCNT solutions are diluted to 20 mL. The shadow indicates the optical absorption peak of (6,4) in first subband. (b) Optical adsorption spectra of the as-prepared SWCNT solutions with the initial concentration of 4 mg/ml using the re-dispersion method and the traditional method followed by centrifugation of 2 hours. (c) Optical absorption spectra of the separated (6, 4) SWCNTs from the individualized SWCNT solutions prepared by re-dispersion method and traditional method with 2-h centrifugation. The eluted SWCNT solutions are diluted to 20 mL. (d) AFM images of the SWCNTs after the first period of sonication (24 h) and ultracentrifugation (1 h) and (e) after the second period of sonication (1 h) and ultracentrifugation (15 min). The height variations along the white dash line in d) and e) were exhibited below the corresponding AFM images. Multiple small bundles with the diameter larger than 1.5 nm were observed in d). While most of the nanotubes shown in e) were less than 1.5 nm in diameter.

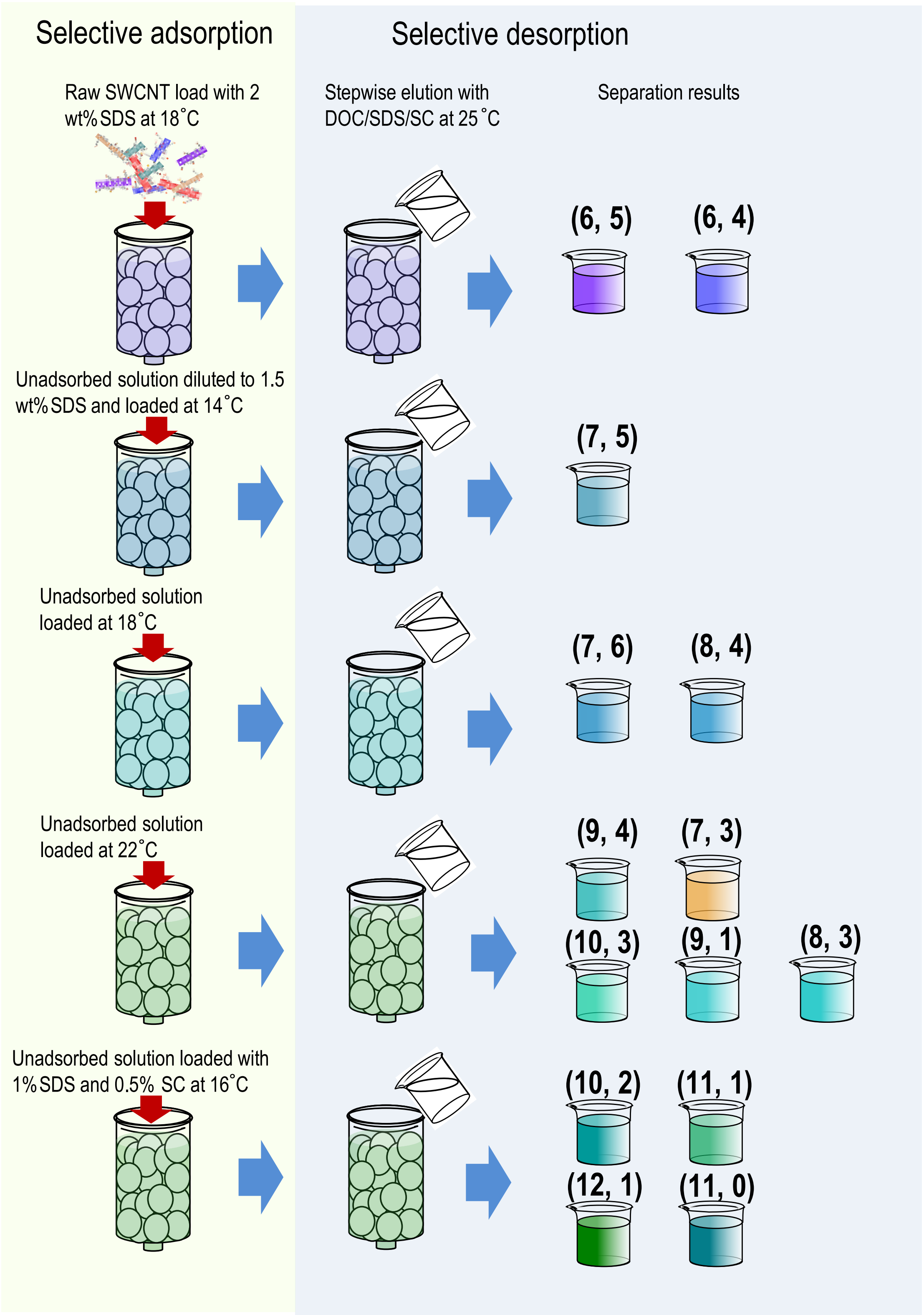

Supplementary Figure 4. Schematic diagram of mass separation procedure of single-chirality species from HiPco-SWNTs.

Supplementary Note 4: Comparison of the previously reported and the current separation strategy

In the previous works[5,6], overloading was employed to achieve the selective adsorption of specific (*n, m*) species at room temperature, in which excessive amount of SWCNTs were loaded to a gel column to promote the competitive adsorption of different (*n, m*) species. In this case, the loaded amount of SWCNTs must be much larger than the capacity of the gel column. Therefore, this method is suitable for small gel columns. Overloading has low resolution on the chiral structure of SWCNTs. To separate single-chirality species, multiple iterative overloading or stepwise elution has to be performed. Meanwhile, many SWCNTs that are capable of being adsorbed flowed through the column and remained in the unsorted solutions, which decreases the utilization rate of raw materials. These characteristics decrease the separation efficiency of SWCNTs and thus hindering the industrial production of single-chirality species.

To further clarifying the characteristics of the overloading method, 10 mL of 4-mg/mL HiPco-SWCNTs dispersed in 2 wt% SDS was loaded to a 10-mL gel column at 25 ºC. The adsorbed SWCNTs were eluted by 5 wt% SDS solution and characterized by optical absorption spectra. The unadsorbed fractions were successively loaded to the next columns. As shown in supplementary Fig. 5a, although most of the (6, 4) and (6, 5) SWCNTs were adsorbed in the first column (Col.1), there was still a considerable amount of them left in the unadsorbed fractions, as evidenced by the adsorption peaks of (6, 4) and (6, 5) in Cols. 2-4. To separate the (6, 4) and (6, 5) in the flow-through fractions, additional separation rounds were required, which significantly degraded the separation efficiency. Besides, because the (6, 4) and (6, 5) that adsorbed in the following columns were relatively lower in purity, their separation efficiency decreased dramatically.

The irreversible adsorption under overloading was investigated. 30 mL of (6, 4)-enriched sample was loaded to a 1-mL gel column equilibrated by 2 wt% SDS. Then, the column was rinsed by 2 wt% and 5 wt% SDS. 35 mL of unadsorbed and 5 mL of eluted SWCNT solutions were collected and characterized by optical absorption spectra,

as shown in supplementary Fig. 5b. Overloading was evidenced by an increase in relative intensity of the (6, 4) absorption peak compared with that of (6, 5) in the eluted solution. The irreversible adsorption of (6, 4) was estimated by $R_{Ir} = \frac{A_{total}*V_{total}-A_{un}*V_{un}-A_{eluted}*V_{eluted}}{A_{total}*V_{total}}$, where A_{un}, A_{eluted} and A_{total} are the area of S_{11} peaks of (6, 4) in optical absorption spectra of the unadsorbed fraction, the eluted fraction and the loaded SWCNTs, V_{un}, V_{eluted} and V_{total} are the volumes of corresponding solutions. Under the overloading condition, the irreversible adsorption is approximately 28%, which is higher than that of the normal loading condition (~ 15% as shown in supplementary Fig. 24d in Supplementary Note 10). Collectively, although higher purity and even enantiomer separation could be achieved through iterative separations under overloading[6], the separation efficiency is much lower compared with our current method.

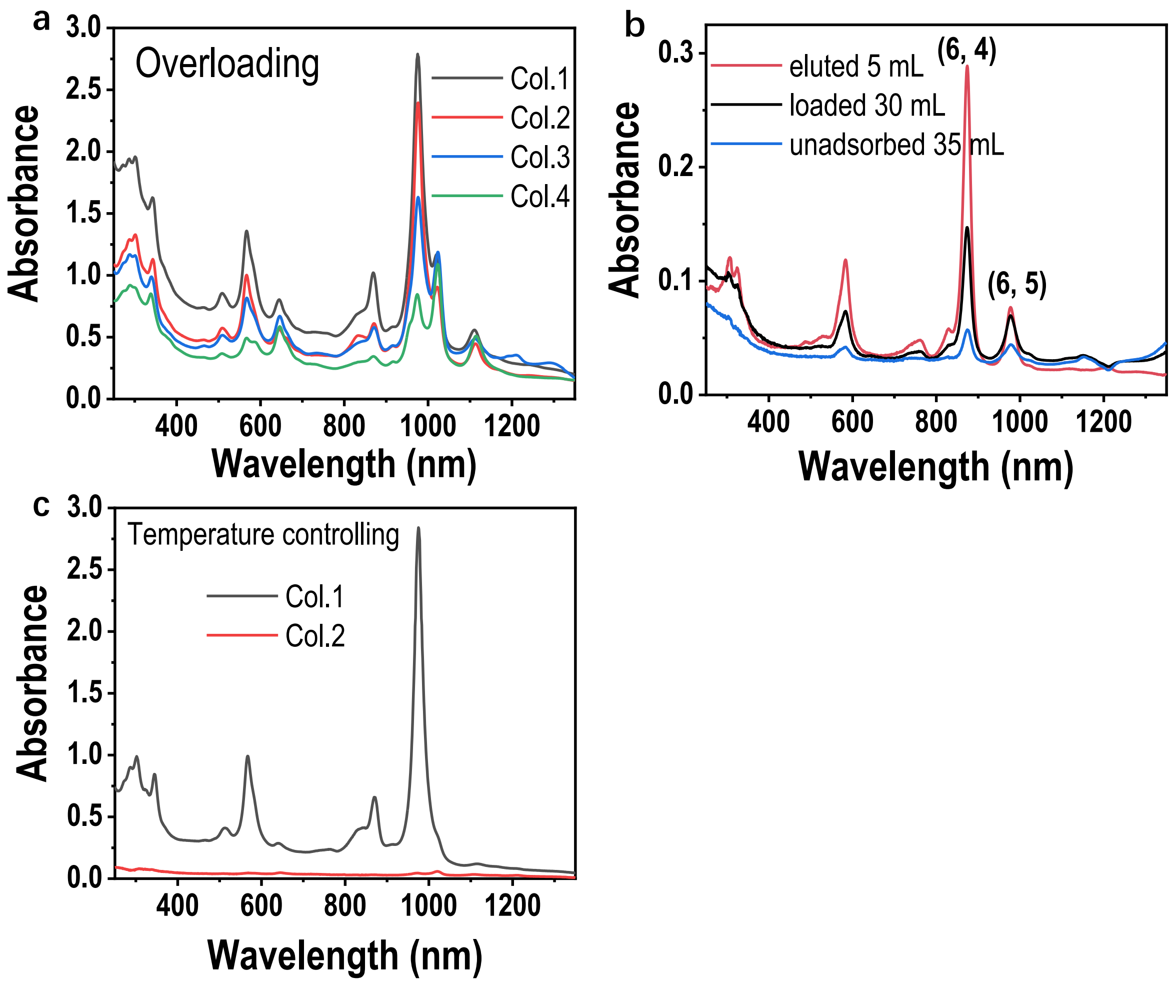

Supplementary Figure 5. Comparison of overloading and temperature-controlled selective adsorption. (a) Optical absorption spectra of eluted SWCNTs in repeated columns under the condition of overloading. The volume of each eluted solution was tuned to 20 mL. (b) Optical absorption spectra of loaded, unadsorbed and eluted SWCNTs. (c) Optical absorption spectra of eluted SWCNTs under normal loading conditions at 18 ºC. Notably, the eluted SWCNT solutions were diluted to 40 mL due to the absorbance intensity exceeding the test range.

For comparison, selective adsorption of SWCNTs was performed by temperature control. 10 mL of HiPco-SWCNTs with an initial concentration of 4-mg/mL was loaded to a 40-mL gel column at 18 ºC. After eluting the unadsorbed SWCNTs, an aqueous solution of 5 %wt SDS was loaded to elute the adsorbed SWCNTs. At a specific temperature, the target (*n, m*) SWCNTs can be selectively adsorbed under normal loading and are fully extracted due to high resolution, and the adsorption of undesired (*n, m*) species was prevented and flow through gel columns. The amount of the adsorbed SWCNTs under a normal loading condition is certainly larger than that under overloading, leading to an increase in the separation efficiency. As exhibited in supplementary Fig. 5c, nearly all (6, 4) and (6, 5) were adsorbed in Col.1 at 18 ºC, as evidenced by the negligible adsorption in Col. 2. These adsorbed (6, 4) and (6, 5) were subsequently separated by stepwise elution. Clearly, temperature control method exhibits higher separation efficiency.

Moreover, compared with the overloading method, the current separation strategy has high resolution. In the previous report[5], the chirality purities of many of the separated SWCNTs such as (8, 3), (8, 4), (9, 4) and (10, 2) were lower than 70%. And near zizag and zizag SWCNTs cannot be achieved. Recently, by temperature control method, SWCNTs were separated successively by diameter and chiral angle using a two-step method[7, 8]. On the basis of these works, the current method was optimized by combining the selective adsorption and selective desorption to produce single-chirality SWCNTs in a single-step process. Most of them show chiral purities higher than 90% including near zigzag (9, 1) SWCNTs.

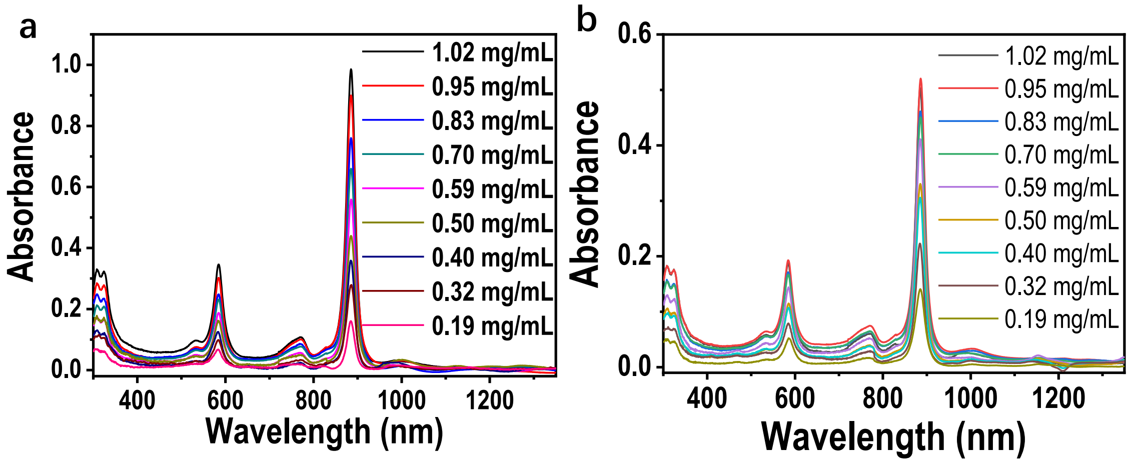

Supplementary Figure 6. The separation yield of (6, 4) SWCNTs under different concentrations of SWCNT solutions. Optical absorption spectra of the separated (6, 4) SWCNTs from HiPco-SWCNT solutions with concentrations ranging from 0.19 to 1.02 mg/ml using a) 40-mL gel columns and b) 10-mL gel columns. Notably, the separation of (6, 4) SWCNTs was performed by selective adsorption at 18 °C and selective desorption at 25 °C, as described in the method section. Specifically, 10 mL of the as-prepared SWCNT dispersion was loaded into a 40-mL gel column.

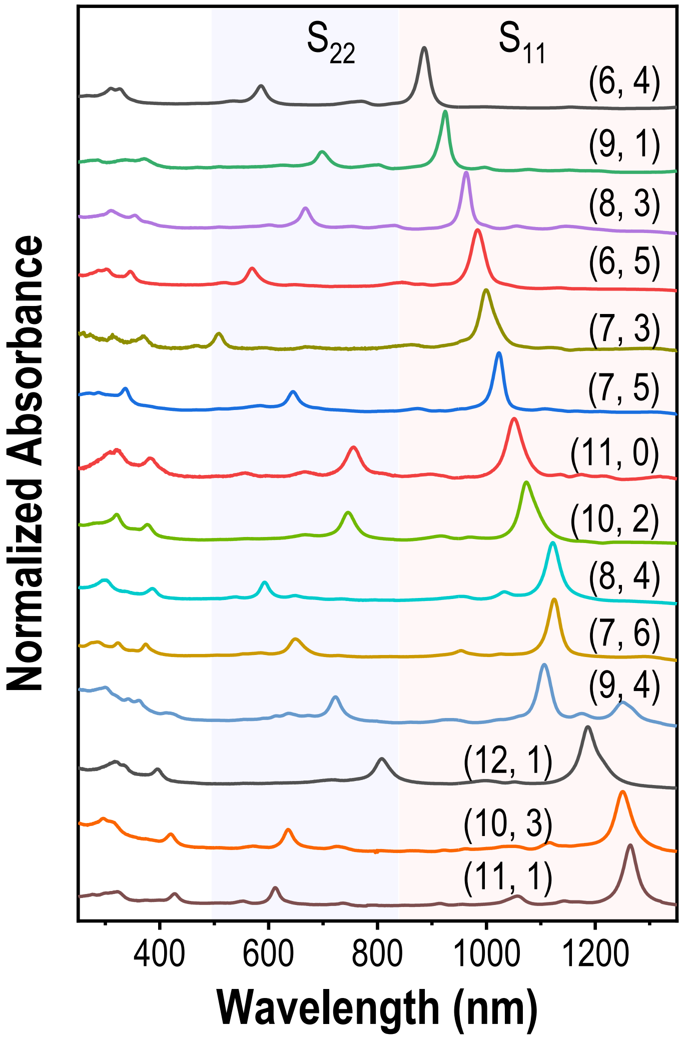

Supplementary Figure 7. Optical absorption spectra of distinct single-chirality SWCNTs separated from high-concentration HiPco-SWCNT solution.

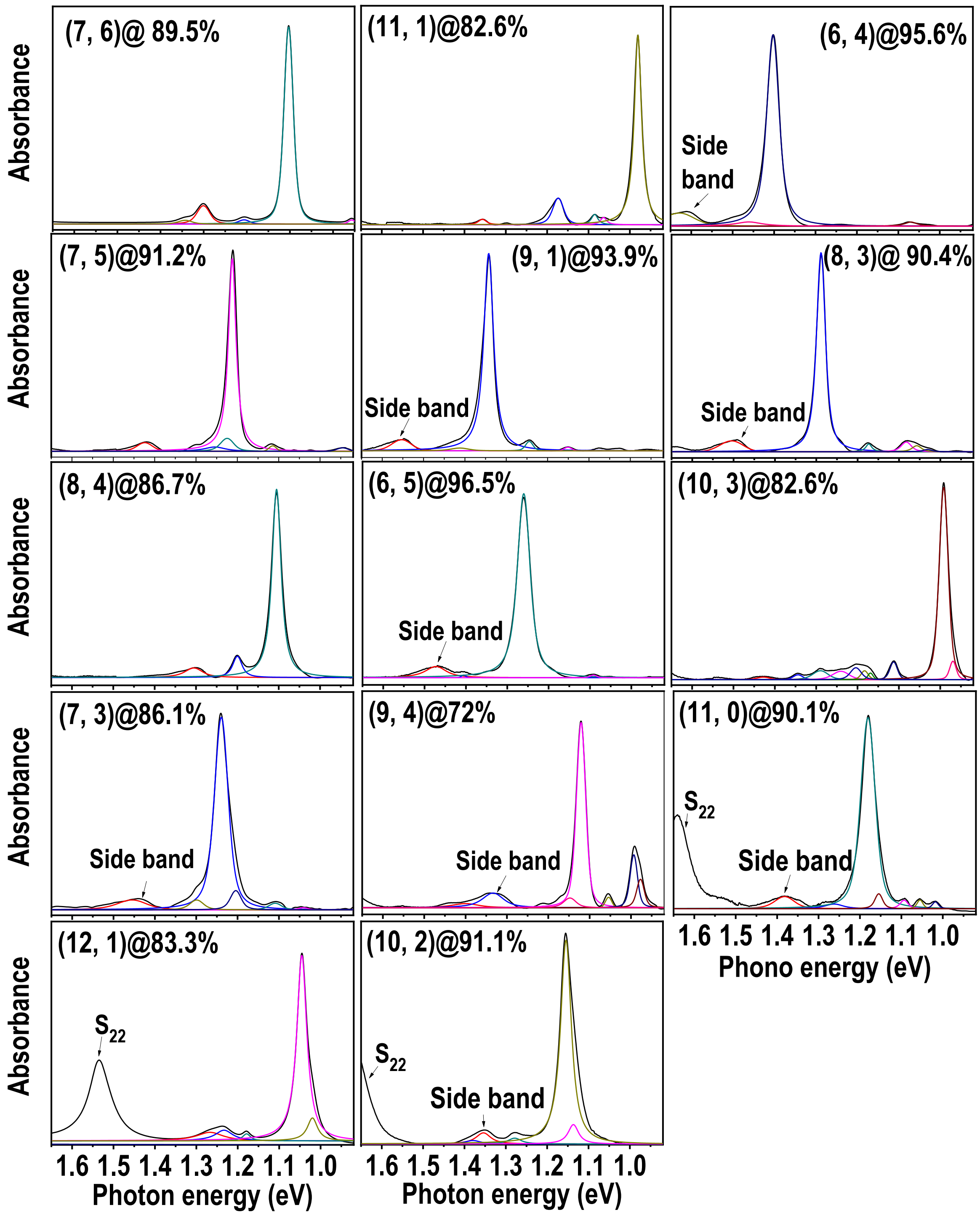

Supplementary Figure 8. Purity evaluation of distinct (*n*, *m*) species separated from HiPco-SWCNT dispersion with initial concentration of 4 mg/mL. The optical adsorption spectrum of each separated (*n, m*) species at the S_{11} region of 800 to 1350 nm were simulated using PeakFit software. The chiral purity was computed as the ratio of the area of the optical absorption peak of the target SWCNT (*n, m*) to the sum of the peak areas: Purity (*n, m*) = Area(*n, m*)/ΣArea(*n, m*).

Supplementary Note 5:

We estimated the amount required to fabricate a large-area SWCNT monolayer on assumption that each SWCNT is well aligned on the substrate. Taking a (6, 5) SWCNT as an example, a 350-nm nanotube in length is composed of ~31000 carbon atoms weighing 6.18×10^{-16} mg. At the linear density of 200 tubes/μm, a 4-inch silicon wafer accommodates 4.64×10^{12} nanotubes weighing 2.87×10^{-3} mg.

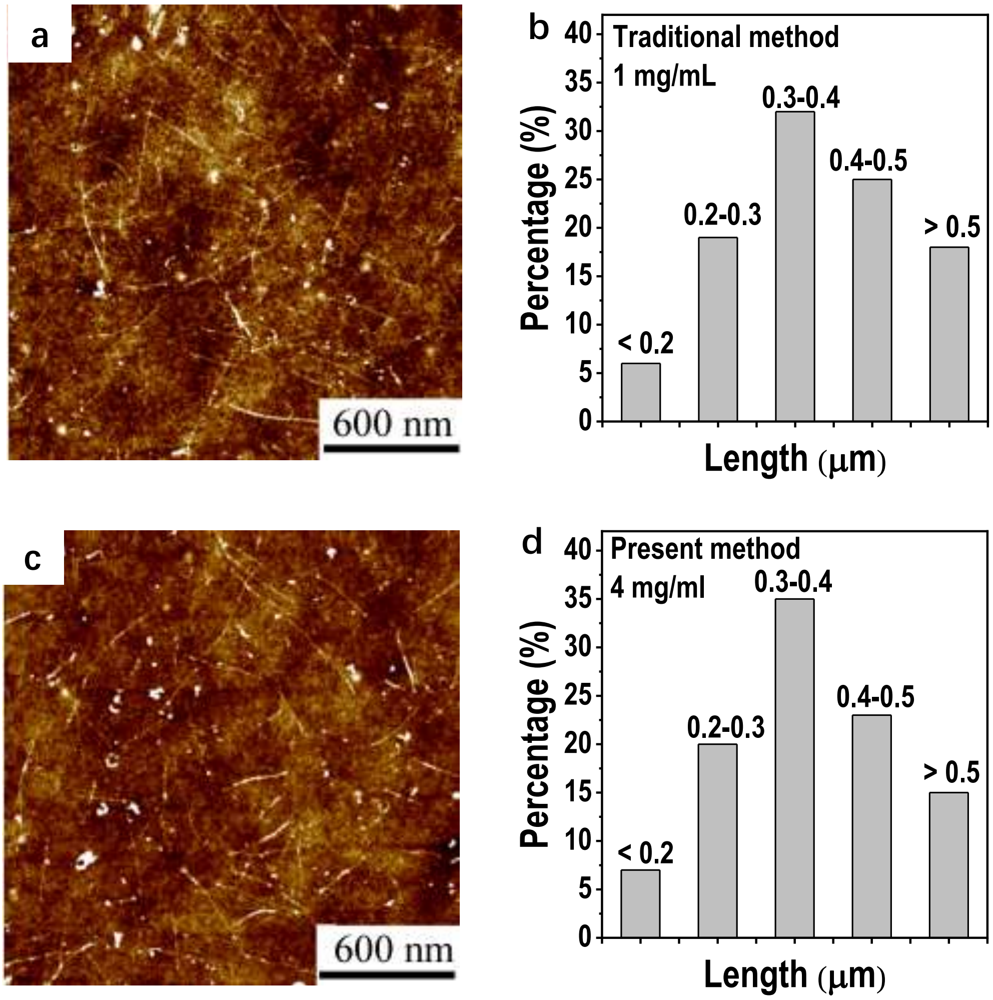

Supplementary Figure 9. **Comparison of length distribution of the as-prepared HiPco-SWCNT dispersions prepared by the conventional and current methods.** (**a**) AFM image of HiPco-SWCNT dispersion with initial concentration of 1-mg/mL prepared by the conventional method: 100 mL of SWCNT dispersion was ultrasonically dispersed for 9 h followed by one-hour ultracentrifugation. (**b**) Length distribution of the nanotubes corresponding to (**a**). (**c**) AFM image of HiPco-SWCNT dispersion with initial concentration of 4 mg/mL prepared by current method. 100 mL of SWCNT dispersion was ultrasonically dispersed for 24 h in the first dispersion stage and for 1 h in the second dispersion stage. (**d**) Length distribution of the nanotubes corresponding to (**c**). The results show that, although the dispersion time of high-concentration individualized SWCNT is longer, the length distribution of the resulted SWCNTs does not change significantly.

Supplementary Note 6: The impact of high-concentration SWCNT solutions on gel column's life time and performance.

Ten-mL SWCNT solutions with the initial concentration of 4 mg/mL were separated by a column filled with 40 mL of gel using the method described in supplementary Note 1. The gel colum was recycled for 20 separation runs. The optical absorption spectra of the separated (6, 4) SWCNTs are presented in supplementary Fig. 10a. Clearly, the purities of the separated (6, 4) SWCNTs were not affected by the recycling of gel, but the varied absorbance indicated that the yield of (6, 4) SWCNTs changed with cycle numner. The relationship between the (6, 4) yield and the number of cycles of gel column were plortted in supplementary Fig. 10b, where the yield of (6, 4) SWCNTs was represented by the S_{11} peak area. Interestingly, the separation yield slightly increased by 8-15% within the first 5 cycles. However, the yield decreased upon 5 cycles. After 20 cycles, the yield decreased by approximate 30% compared with the new gel in a separation round. This result was confirmed by three independent experiments. The yield variation of (6, 4) SWCNTs should be strongly realted to the irreversible adsorption of SWCNTs in the gel column. Due to the presence of a large number of irreversible adsorption sites in the new gel, the loaded SWCNTs suffered from loss due to irreversible adsorption. With increasing the number of cycles, the amount of irreversible adsorption possibly decreased due to the occupation of irreversible adsorption sites as discussed in supplemenatry Note 10. Therefore, within the first five cycles, the production of (6, 4) SWCNTs increased. With continously increasing the number of cycles, the reversible adsorption sites may begin to turn into irreversible adsorption sites, weakening the adsorption capacity of the gel column and thus a decrease in the separation yield. Additionally, the impurities such as amorphous carbon are also likely to occupy adsorption sites and form irreversible adsorption, thus decreasing the gel adsorbability to SWCNTs[9]. The degradation of gel may vary with the type of surfactant, the structural distribution of SWCNTs, separation temperature and the dispersity of SWCNT solutions.

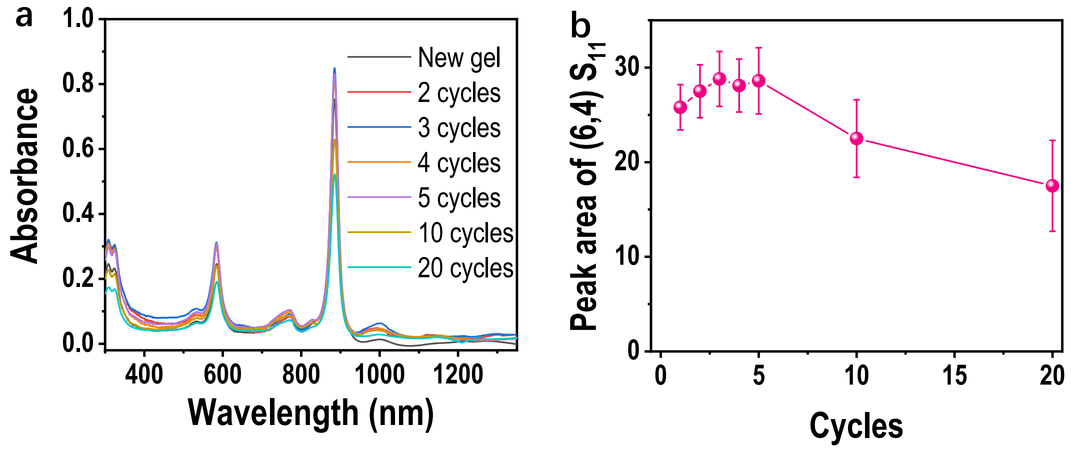

Supplementary Figure 10. The variation of gel performance with respect to cycles. (a) Optical absorption spectra of the separated (6, 4) using recycled gel. (b) Relationship between the (6, 4) yield and the number of cycles of gel column. The amount of collected (6, 4) was evaluated by the S_{11} peak area of (6, 4) in the range from 800 to 940 nm. Error bars in (b) are the standard deviation of statistics by repeating the experiment for three times.

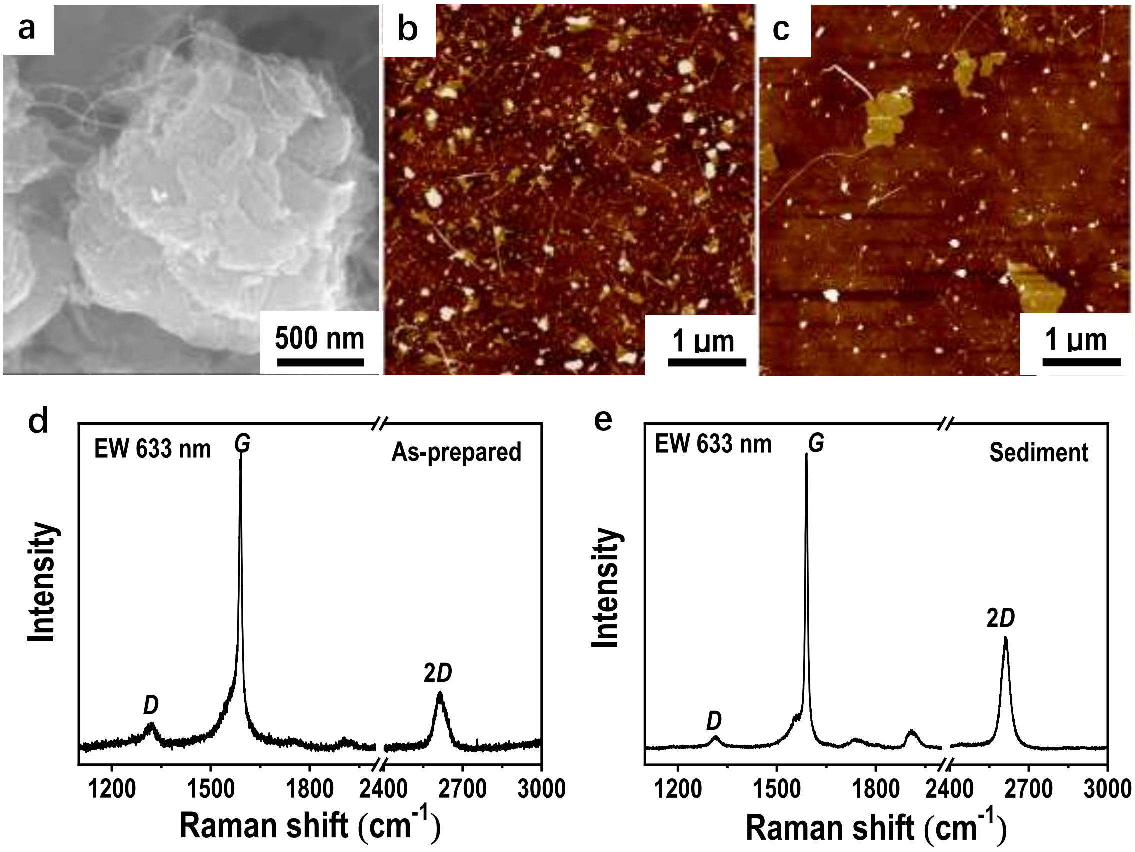

Supplementary Figure 11. Characterization of the G-SWCNT dispersion.

(a) SEM image of the raw G-SWCNTs. (b) AFM image of the as-prepared G-SWCNT dispersion with an initial concentration of 4 mg/mL. (c) AFM image of centrifugal precipitate in the first centrifugation. (d) Raman spectrum corresponding to the samples in b). (e) Raman spectrum corresponding to the samples in c). EW is the abbreviation of excitation wavelength.

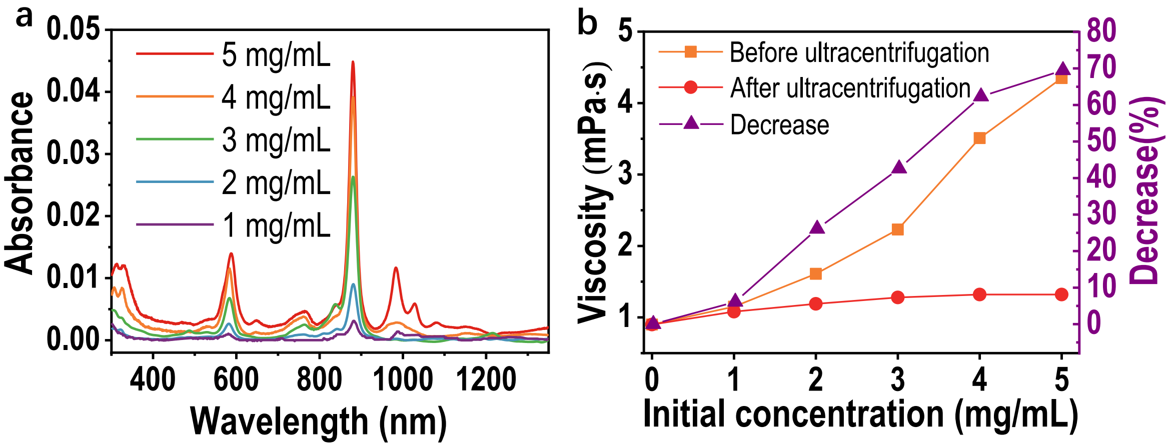

Supplementary Figure 12. Characterization of the dispersity and viscosity of high-concentration G-SWCNT dispersion.

a) Optical absorption spectra of (6, 4) SWCNTs by loading 10 mL of G-SWCNT dispersion with different initial concentrations into a gel column of 40 mL; The separated (6, 4) were diluted to 30 mL for comparison of their yield. b) The shear viscosity of the as-dispersed SWCNT solution before and after centrifugation as a function of initial concentrations. After ultracentrifugation, the viscosity of the resulted supernatant decreases, but higher than that of HiPco-SWCNTs with the same initial concentration, possibly because of the presence of small graphene segments (Supplementary Fig. 11).

Supplementary Table 1. Peak area of S_{11} of different (*n*, *m*) species in Figure 3e

	(6, 4)	(9, 1)	(6, 5)	(7, 3)	(7, 5)	(9, 4)	(8, 4)	(7, 6)	(10, 3)
**1 mg/mL**	0.17	0.13	—	—	—	0.25	—	—	0.32
**2 mg/mL**	0.42	0.39	0.51	0.57	0.52	0.65	0.66	0.64	0.92
**4 mg/mL**	1.91	1.65	2.50	2.46	2.18	2.77	3.03	2.60	3.67

Note: Peak areas of S_{11} were calculated by integrating the absorbance from 800 nm to 1350 nm.

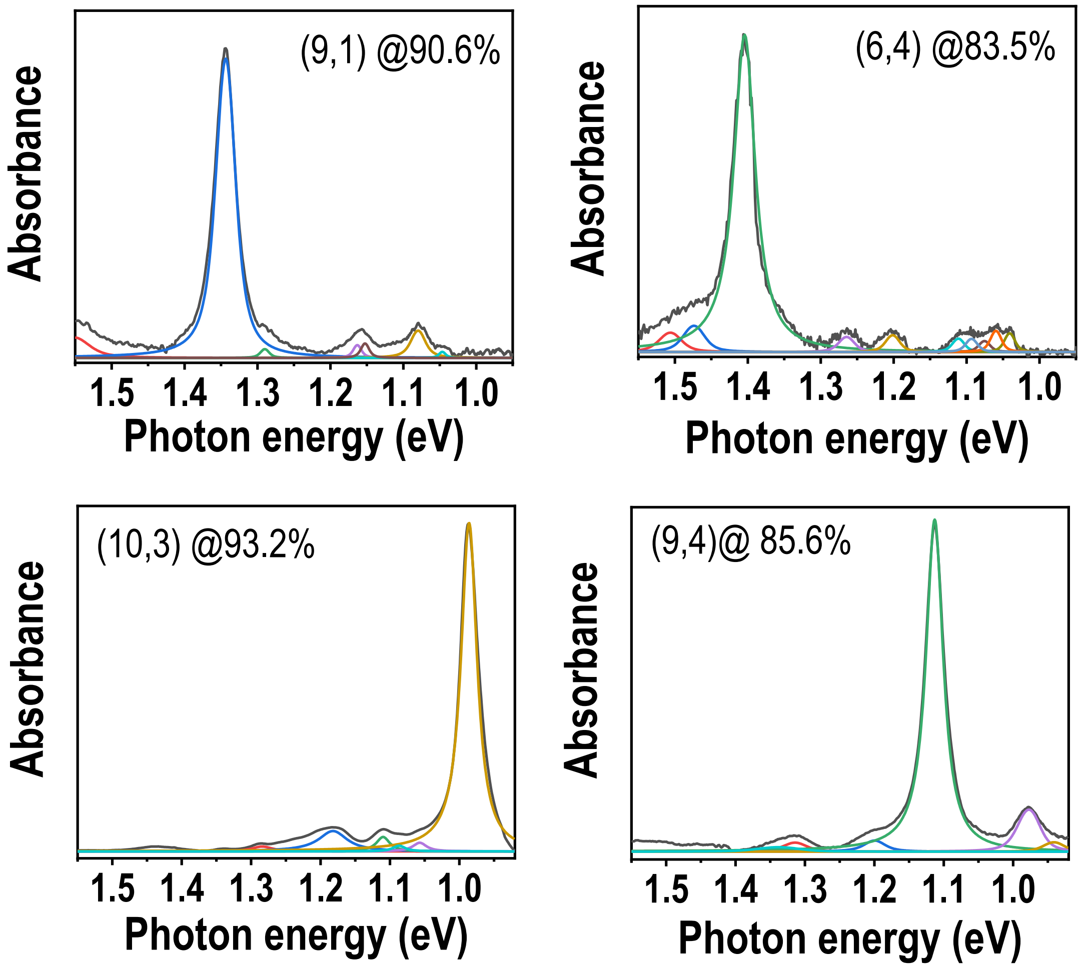

Supplementary Figure 13. **Purity evaluation of the (*n, m*) species separated from G-SWCNTs with the initial concentration of 1 mg/mL.**

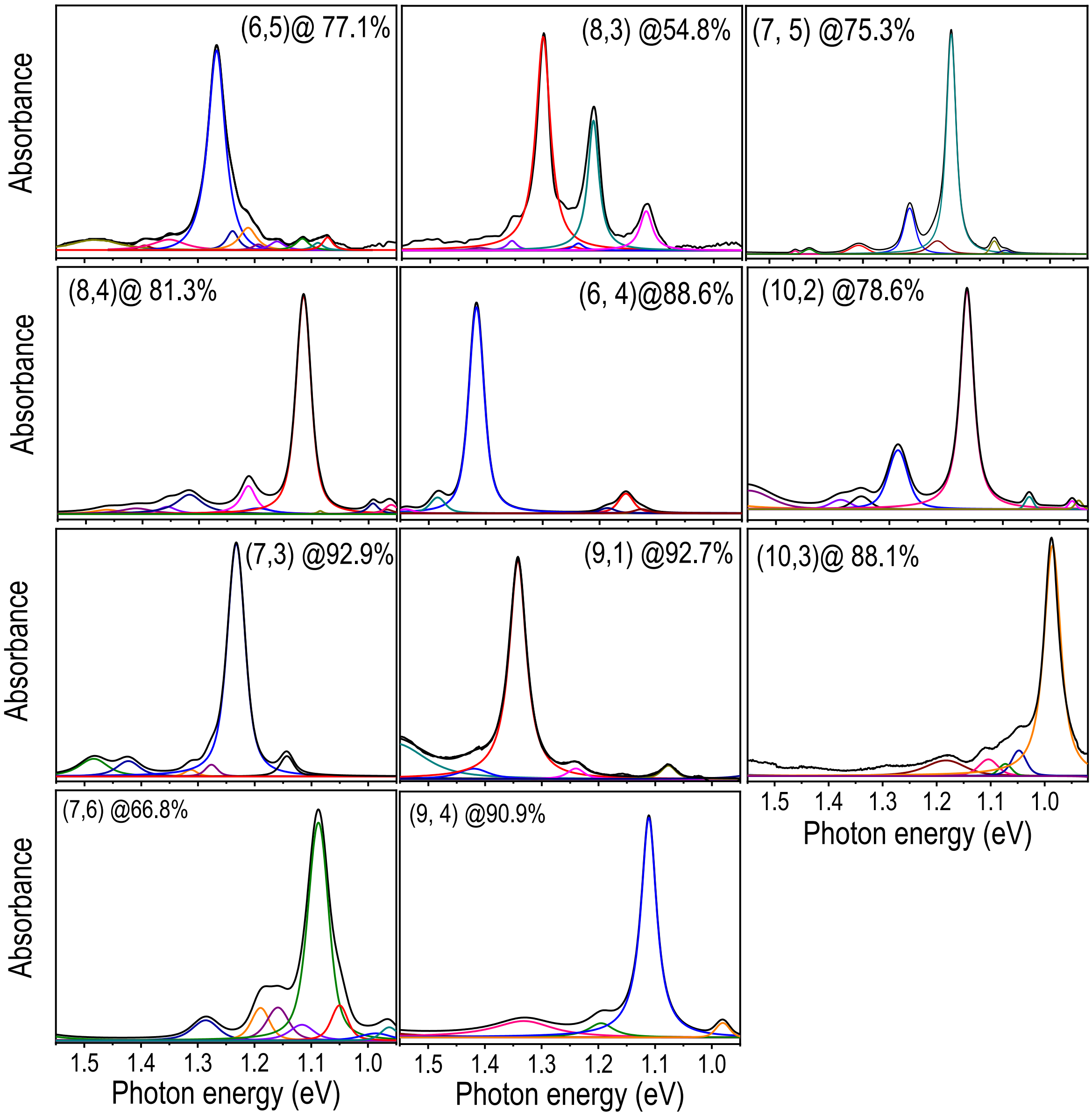

Supplementary Figure 14. **Purity evaluation of the (*n, m*) species separated from G-SWCNTs with the initial concentration of 4 mg/mL.** The optical adsorption spectrum of each separated (*n, m*) species at the S_{11} region of 800 to 1350 nm were simulated using PeakFit software. The chiral purity was computed as the ratio of the area of the optical absorption peak of the target SWCNT (*n, m*) to the sum of the peak areas: Purity (*n, m*) = Area(*n, m*)/ΣArea(*n, m*).

Supplementary Note 7: Comparison of the absolute contents of semiconducting SWCNTs with diameters range of 0.8-1.2 nm in G-SWCNTs and HiPco-SWCNTs.

To compare the contents of SWCNTs with diameter range of 0.8-1.2 nm in the two raw materials, 80 mL of G-SWCNT individualized solution with initial concentration of 1 mg/mL were dispersed in aqueous solution of 0.5 wt % SDS and then loaded into a gel column of 40 mL at 25 °C. The adsorbed SWCNTs were eluted with aqueous solution of 5 wt % SDS and the volume was tuned to 20 mL for the measurement of optical absorption spectra. In contrast, 4.5 mL of HiPco-SWCNT solution with initial concentration of 1 mg/mL was diluted to 80 mL with 0.5 wt% SDS solution and loaded into the same gel column. The adsorbed SWCNTs were eluted and diluted to 20 mL for comparison. The corresponding optical absorption spectra show that the absorbance at 273 nm of the semiconducting SWCNTs extracted from the two raw materials are approximately equal in amount (Supplementary Fig. 15). As indicated in the main text, an individualized SWCNT solution of 0.19 mg/mL could be obtained by dispersing HiPco-SWCNTs at an initial concentration of 1 mg/mL. From this, it was calculated that the concentration of small-diameter SWCNTs in G-SWCNTs with an initial concentration of 1 mg/mL should be approximately 0.011 mg/mL. At the same time, the optical absorption spectra show that the chiral types of SWCNTs in the two raw materials are different, the relative contents of smaller-diameter SWCNTs are smaller. The optical absorption spectra of the selectively adsorbed SWCNTs in the case of 1.5 wt% at 18 ºC exhibit clearer difference (Supplementary Fig. 16). The relative contents of (6, 5), (8, 3) and (7, 3) SWCNTs in G-SWCNTs is smaller. This may be why the purity of (8, 3) SWCNTs separated from G-SWCNTs is relatively lower.

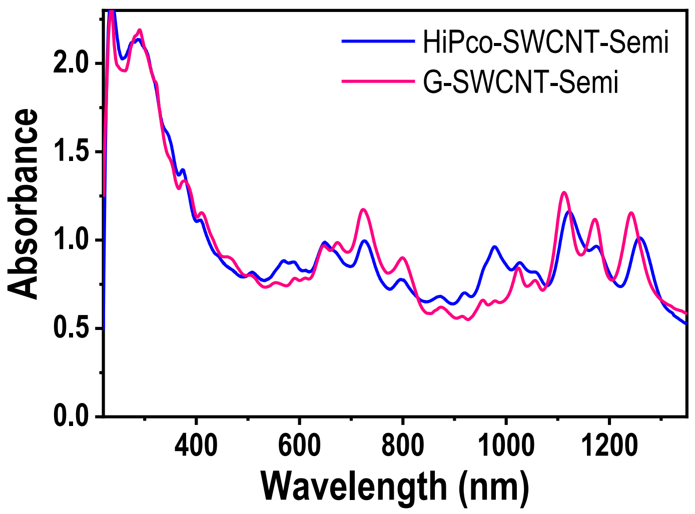

Supplementary Figure 15. **Optical absorption spectra of the SWCNTs selectively adsorbed in gel column from G-SWCNTs and HiPco-SWCNTs dispersed in aqueous solution of 0.5 wt % SDS.** 80 mL of individualized G-SWCNT solution with initial concentration of 1 mg/mL is loaded into a gel column of 40 mL; 4.5 mL of HiPco-SWCNT solution with initial concentration of 1 mg/mL was diluted to 80 mL and loaded into the same gel column.

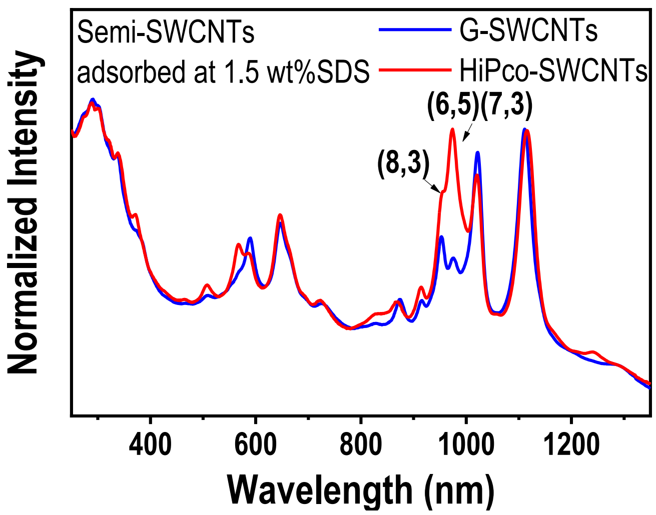

Supplementary Figure 16. **Normalized optical absorption spectra of SWCNTs selectively adsorbed into gel column from G-SWCNTs and HiPco-SWCNTs dispersed in aqueous solution of 1.5 wt % SDS at 18 °C.** The relative optical absorbances of (6, 5), (7, 3) and (8, 3) in G-SWCNTs are distinctly smaller than those in HiPco-SWCNTs.

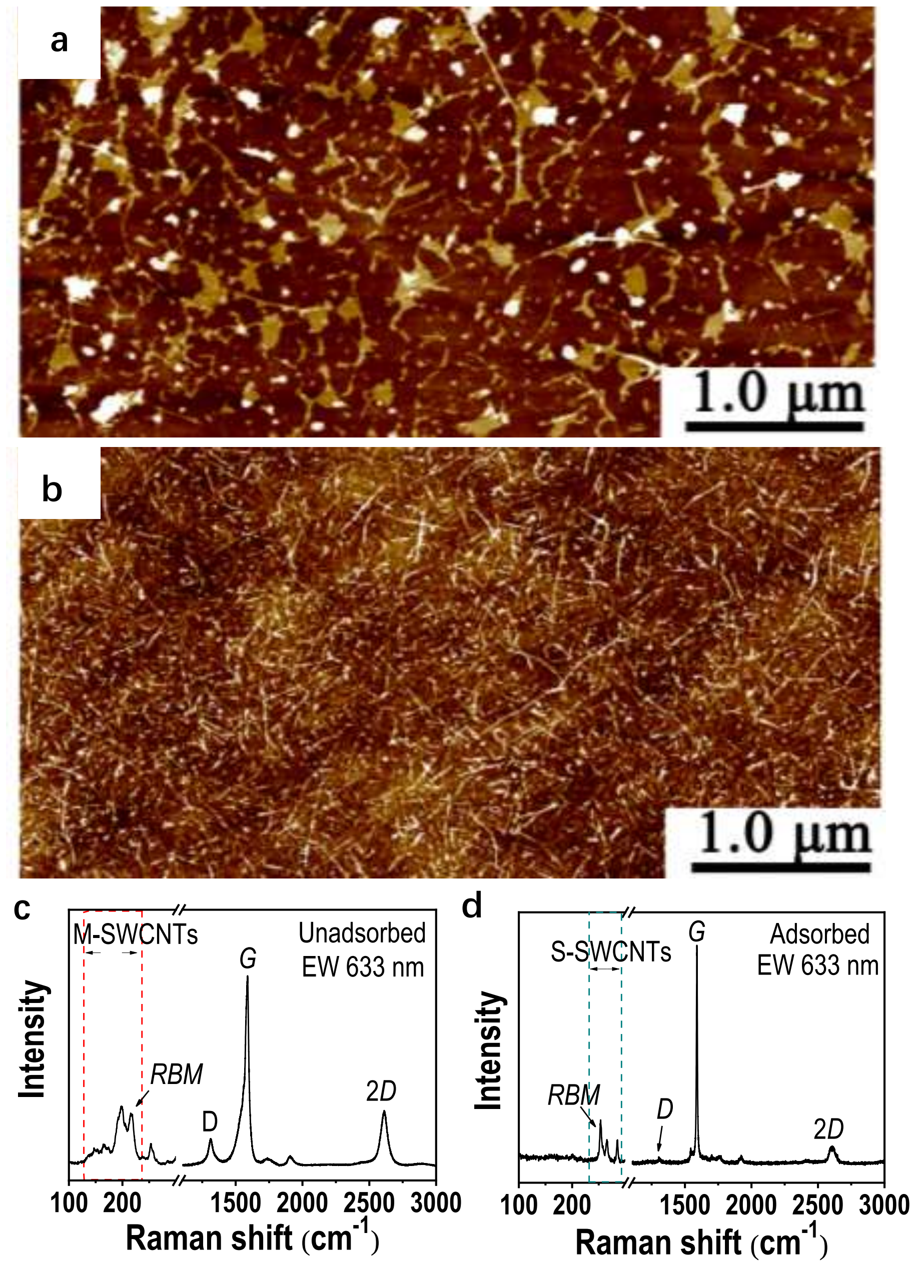

Supplementary Figure 17. Characterization of the unadsorbed and adsorbed SWCNTs in the gel column when the as-prepared G-SWCNT dispersion was loaded. a) and b) AFM images of unadsorbed and adsorbed G-SWCNTs in the gel columns. c) and d) Raman spectra of the corresponding unadsorbed and adsorbed SWCNTs. M-SWCNTs and S-SWCNTs represent metallic and semiconducting signle-wall carbon nanotubes. EW is the abbreviation of excitation wavelength. Clearly, the graphene or graphite in the G-SWCNT dispersion is unable to be adsorbed onto gel column in present separation parameters, and directly flowed through gel columns with metallic SWCNTs. The adsorbed SWCNTs are semiconducting SWCNTs. Their Raman

spectra show a large G/D ratio, indicating that they have high crystallinity.

Supplementary Note 8: Mass separation of large-diameter semiconducting SWCNTs from high-concentration G-SWCNT dispersion.

The separation of large-diameter semiconducting SWCNTs was followed the previous method[9]. The separation procedure is as follows: Individualized G-SWCNT solution of 300 mL was prepared by ultrasonically dispersing raw G-SWCNT solution with the initial concentration of 4 mg/mL in an aqueous solution of 0.5 wt% SDS. Semiconducting SWCNTs with small diameters were extracted by loading the raw materials into a column filled with 160 mL of gel at 28 °C. The rest of nanotubes were difficult to be adsorbed. Therefore, NaOH was introduced into the SWCNT solution to tune the adsorbability of SWCNT into the gel medium. The concentration was varied from 0 to 0.5 mM with an increase step of 0.1 mM. The adsorbed nanotubes were eluted with an aqueous solution of 5 wt % SDS without NaOH. Subsequently, the NaOH concentration in the flow-through fraction was increased to a higher concentration for the next round using the same procedure.

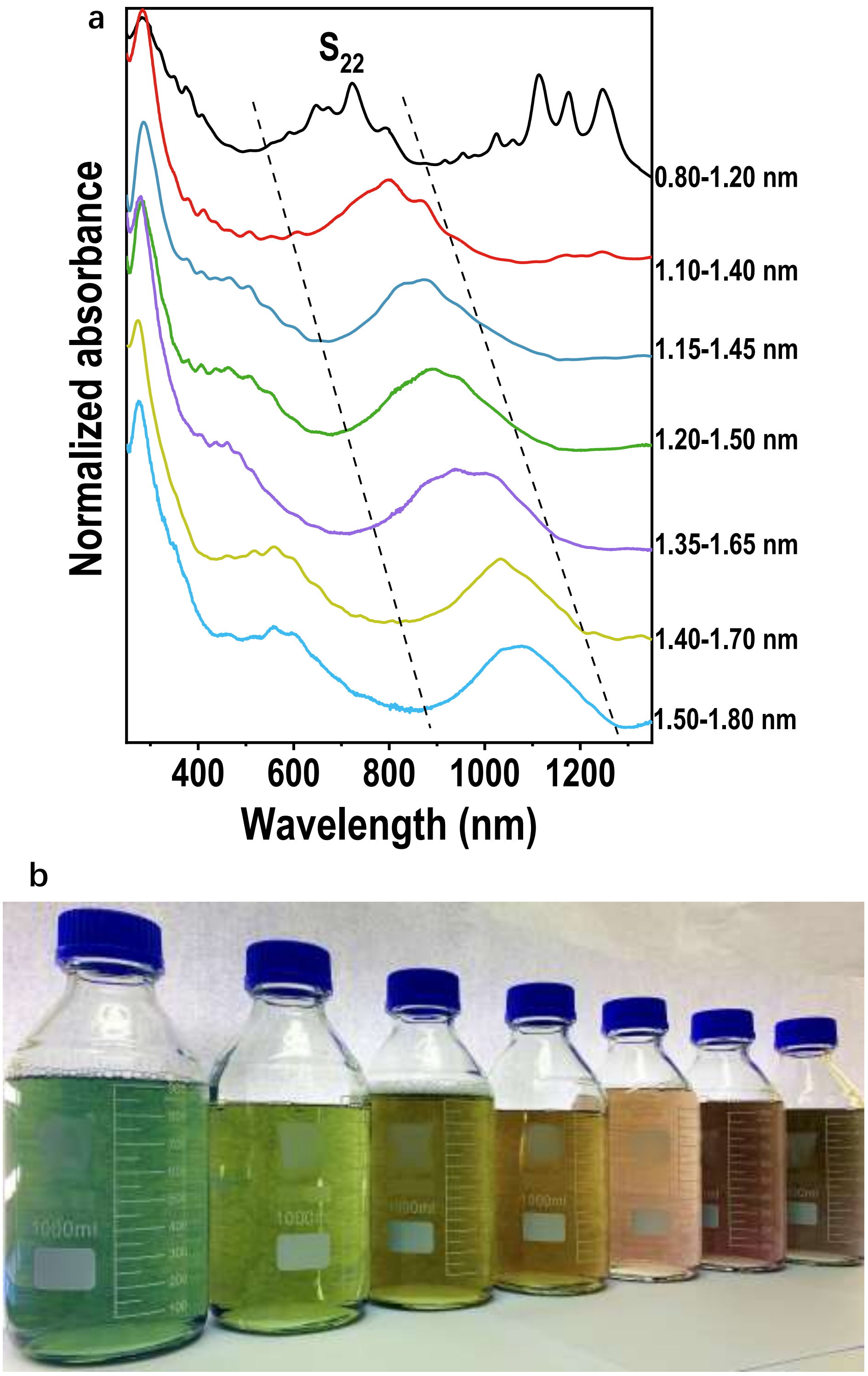

Supplementary Figure 18. Characterization of the semiconducting SWCNTs with different diameters industrially separated from a high-concentration individualized G-SWCNT solution. (a) Optical adsorption spectra of the

semiconducting SWCNTs separated from G-SWCNTs. (b) Solution photographs of the separated SWCNTs with different diameters: From left to right, the diameter ranges are 0.80-1.20 nm, 1.10- 1.40 nm, 1.15-1.45 nm, 1.20-1.50 nm, 1.35-1.65 nm, 1.40-1.70 nm and 1.50-1.80 nm, which are assigned according to their S_{22} peaks, respectively[9, 10].

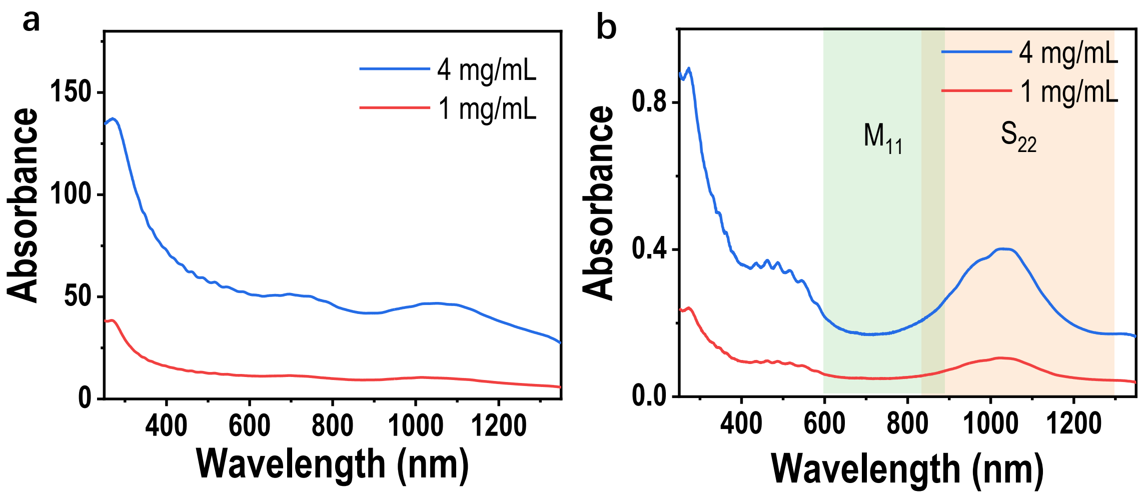

Supplementary Figure 19. Preparation of high-concentration Tuball SWCNT solution. (a) Optical absorption spectra of as-prepared Tuball SWCNT solutions with different initial concentrations. The preparation method is similar with that of G-SWCNTs. (b) Optical absorption spectra of semiconducting SWCNTs separated from Tuball SWCNT solutions. The semiconducting SWCNTs were adsorbed at 0.25 wt% SC/ 0.5 wt% SDS at 25 ºC. The absence of M_{11} peaks indicates that the separated semiconducting SWCNTs have high semiconducting purity, implying a high dispersity of the as-prepared SWCNT solution. The eluted semiconducting SWCNTs were diluted to 40 mL for comparison. The difference in absorption absorbance suggests the yield of semiconducting SWCNTs increased by approximate 4 times when using Tuball SWCNT solution with an initial concentration of 4-mg/mL.

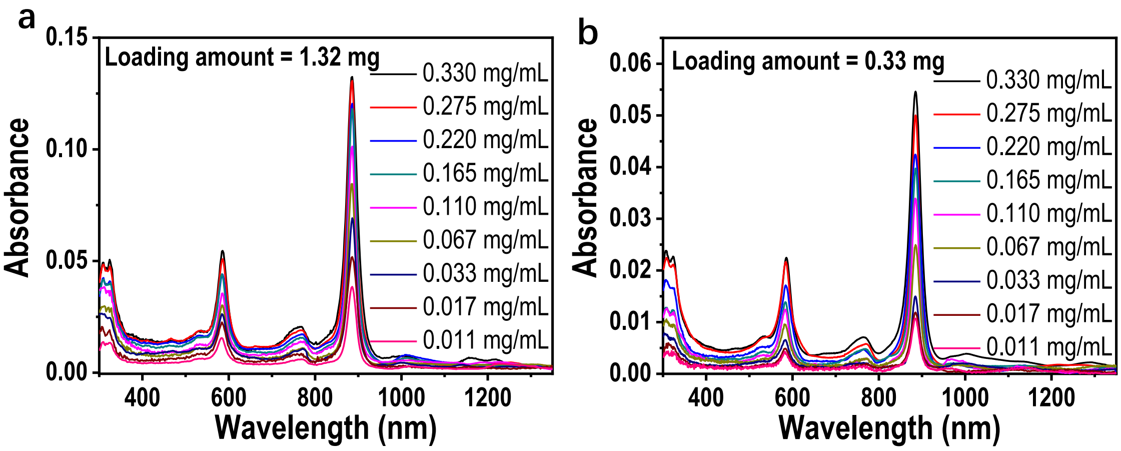

Supplementary Figure 20. Effect of concentration of SWCNT dispersion on the separation yield of (6, 4) SWCNTs. (a) and (b) Optical adsorption spectra of the separated (6, 4) species under different HiPco-SWCNT concentrations with fixed loading amounts of 1.32 mg and 0.33 mg. Specifically, HiPco-SWCNTs of 1.32 and 0.33 mg with different concentrations were loaded to columns of 40 mL at 18 °C. After eluting the unadsorbed SWCNTs with 2 wt% SDS at 18 °C, the surfactants in column were replaced with 0.5 wt% SDS/ 0.5 wt% SC, during which the separation temperature was increased to 25 °C. The (6, 4) SWCNTs in the gel columns were eluted by the cosurfactant solution of 0.025 wt% DOC/0.5 wt% SC/0.5 wt% SDS. The volumes of the separated (6, 4) SWCNTs under different loading amounts were tuned to 20 mL for comparison.

Supplementary Note 9: The effect of the concentration of the loaded SWCNTs on the proportions of the reversibly adsorbed, unadsorbed and irreversibly adsorbed SWCNTs in gel chromatography.

In the separation process of SWCNTs by gel chromatography, the SWCNTs loaded into the gel column were separated into reversibly adsorbed, unadsorbed and irreversibly adsorbed SWCNTs. The increase in the separation yield of single-chirality SWCNTs caused by loading a high-concentration of individualized SWCNT solution should be induced by the relative changes of the three parts mentioned above. To clarify the mechanism of the yield increase in the separation of single-chirality SWCNTs with high-concentration individualized raw SWCNT solution, it is necessary to systematically study the influence of the concentration and loading amount of SWCNTs on the reversible adsorption, unadsorption and irreversible adsorption parts.

To exclude the influence from different types of species and clearly clarify the proportion relationship between reversibly adsorbed, irreversibly adsorbed and unadsorbed SWCNTs, single-chirality (6, 5) SWCNTs of 40 mL dispersed with an aqueous solution of 2 wt% SDS was used as raw materials, which were divided equally into 4 parts and diluted to different concentrations (as shown in supplementary Fig. 21a). Subsequently, each SWCNT solution was loaded into a gel column filled with 40 mL of gel at 25 °C. Then, the unadsorbed SWCNTs were eluted by 0.5 wt% SDS and subsequently by 0.5 wt% SDS/ 0.5 wt% SC solutions. The reversibly adsorbed SWCNTs were eluted by 5 wt% SDS solutions. In each case, the eluted and unadsorbed nanotube solutions were diluted to 30 mL and 100 mL, respectively. The corresponding optical absorption spectra are shown in supplementary Figs. 21b and c, respectively.

The proportion of the unadsorbed SWCNTs was calculated as: $R_{un} = \frac{A_{un}*V_{un}}{A_{total}*V_{total}}$, where A_{un} and A_{total} are the area of S_{11} peaks of (6, 5) in the absorption spectra of the unadsorbed fraction and the loaded SWCNTs, V_{un} and V_{total} are the volumes of corresponding solutions. The proportion of the reversible adsorption was calculated as: $R_{re} = \frac{A_{eluted}*V_{eluted}}{A_{total}*V_{total}}$, where A_{eluted} and V_{eluted} are the optical absorbance and volumes of the eluted fractions. The proportion of the irreversibly adsorbed was

calculated as: $R_{Ir} = \frac{A_{total}*V_{total} - A_{un}*V_{un} - A_{eluted}*V_{eluted}}{A_{total}*V_{total}}$. As shown in supplementary Fig. 21d, the proportion of the irreversible adsorption decreases slightly with decreasing SWCNT concentration under such a fixed loading amount, while the proportion of unadsorbed SWCNTs increased rapidly.

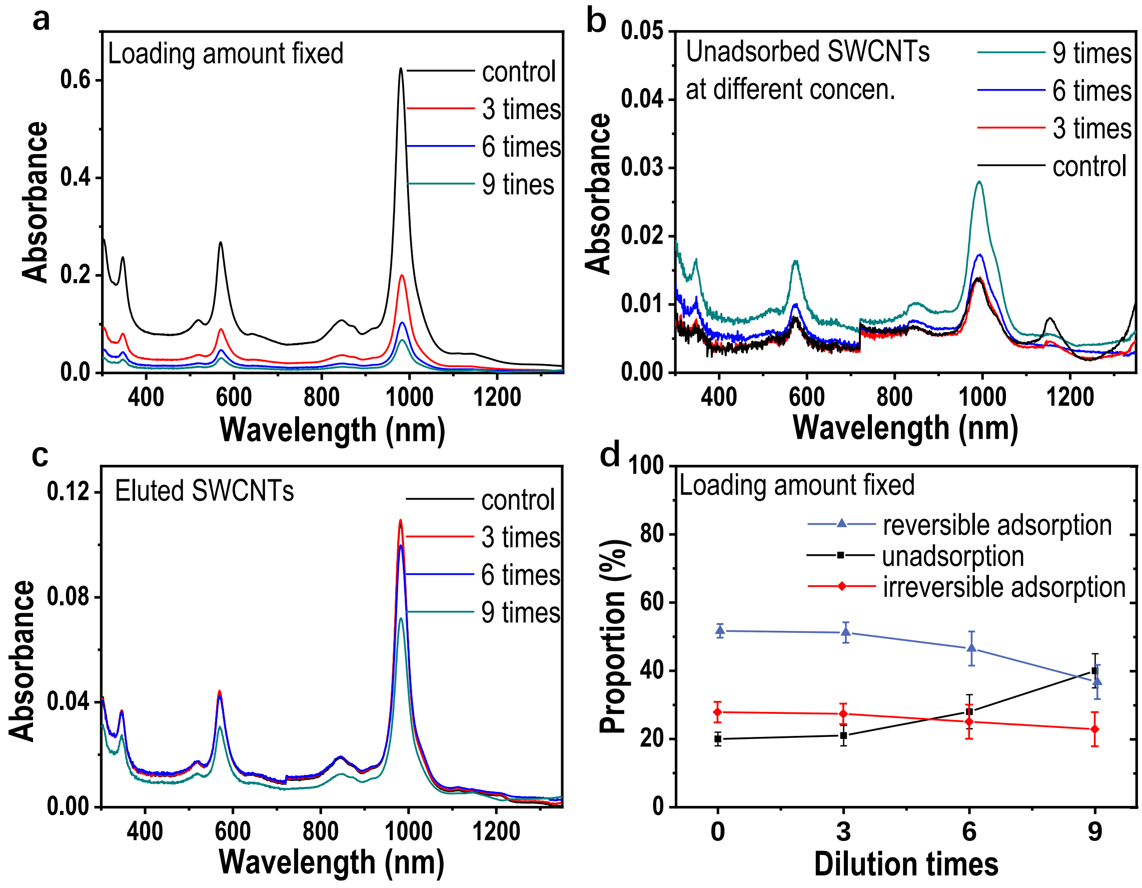

Supplementary Figure 21. **Relationship between the SWCNT concentration and the unadsorption, reversible and irreversible adsorption.** (a)The optical absorption spectra of (6, 5) SWCNT solutions with different concentrations. (b) the unadsorbed SWCNTs and (c) reversibly adsorbed SWCNTs. The legend represents the dilution times of the parent SWCNT solutions. (d) The proportions of the unadsorbed, reversibly and irreversibly adsorbed SWCNTs as a function of the concentrations of the loaded SWCNTs. Error bars in (d) are the standard deviation of statistics by repeating the experiment for three times.

Similar to biomolecules [10], the adsorption process of SWCNTs to gel can be

described as follow:

$$SWCNT_{n,m}^{free} + \theta \xrightarrow{K_{n,m}^{f}} SWCNT_{n,m}^{ads} \quad (1)$$

where $SWCNT_{n,m}^{free}$ and $SWCNT_{n,m}^{ads}$ represent the number of free (*n, m*) species and the number of adsorbed (*n, m*) species in a reaction volume of gel, respectively. θ is the total number of adsorption sites in this area. $K_{n,m}^{f}$ represent the forward rate constants, which is determined by the interaction between a (*n, m*) SWCNT and an unoccupied binding site[11]. At low concentrations, the number of each (*n, m*) species in the reaction volume is low, leading to a low binding rate. The change of concentration of (*n, m*) species with time reveal the binding rate[11]:

$$-\frac{dC_{n,m}(t)}{dt} = K_{n,m}^{f}\, C_{n,m}(t)[\frac{\theta}{V} - \Sigma\left(C_{n,m}(t_0) - C_{n,m}(t)\right)] \quad (2)$$

At a given time, the rate of change of $C_{n,m}(t)$ nonlinearly decreases with $C_{n,m}(t)$. It suggests that the binding of SWCNTs become harder as the concentration decreases.

As shown in supplementary Fig. 22, in a given SWCNTs/gel system, the SWCNT solution can be divided into bulk solution and boundary layer adjacent to gel surface. Due to the fast adsorption of SWCNTs, the concentration of SWCNTs in boundary layer $C_{n,m}^{*}$ is smaller than that in the bulk solution $C_{n,m}$, forming a concentration gradient, which drives transfer of SWCNTs from bulk solution to boundary layer.[12, 13] The simplest expression of the relationship between the flux of a (*n, m*) species and the "driving force" of mass transfer is as follows[10]:

$$J = D_{n,m}(C_{n,m} - C_{n,m}^{*})/\delta \quad (3)$$

where $D_{n,m}$ is the diffusion coefficient of the specific SWCNT/surfactant hybrid[13], δ is the thickness of boundary layer determined by the flow rate of the solution and geometry of gel beads. At low concentration, due to the small difference in the concentrations of $C_{n,m}$ and $C_{n,m}^{*}$, small concentration gradient hinders the mass transfer of SWCNTs to the gel surface and thus decreasing their adsorption onto gel surfaces due to low concentration in the reaction volume adjacent to the gel surface. SWCNTs tend to remain in the bulk solution and may flow through the gel, leading to more unadsorbed SWCNTs, as shown by the yellow line in supplementary Fig. 22.

As the concentration in the bulk solution increases, the difference between $C_{n,m}$ and $C^*_{n,m}$ increases. The transfer of SWCNTs to the gel surface has been enhanced, providing more SWCNTs for the binding reactions, as shown by the red line in supplementary Fig. 22, which reduce the proportion of unadsorbed SWCNTs. Then, as excessive SWCNTs were transferred to the gel surface in a short time, the adsorption kinetics turn to adsorption-control. The SWCNTs adjacent to the gel surface accumulate, which increase $C^*_{n,m}$ and decreases the concentration gradient in the boundary layer. At this stage, the resistance of SWCNT binding is mainly attributed to the adsorption process (red line in supplementary Fig. 22). Therefore, further increasing the SWCNTs concentration, the adsorption amount of SWCNTs tend to saturate. Notably, this model is very simple and does not involve interactions between different (*n, m*) species and SWCNT transfer inside gel beads.

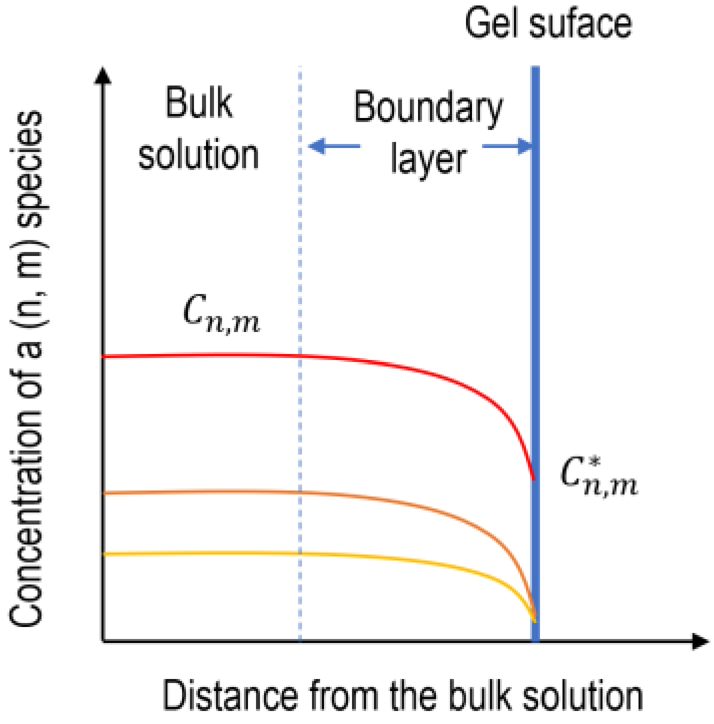

Supplementary Figure 22. Schematic diagram of mass transfer from the bulk SWCNT solution to gel surface.

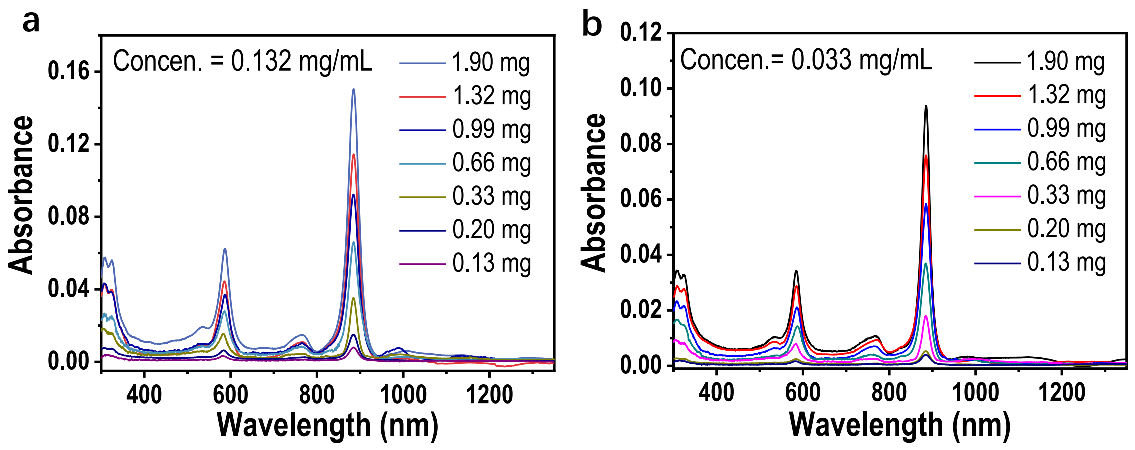

Supplementary Figure 23. Effect of loading amount of SWCNT dispersion on the separation yield of (6, 4) SWCNTs. (a) and (b) Optical adsorption spectra of the (6, 4) species separated by loading different amounts of HiPco-SWCNT dispersion with fixed concentration of 0.132 and 0.033 mg/mL. The volumes of the separated (6, 4) SWCNTs were controlled at 20 mL. Concen is the abbreviation of concentation.

Supplementary Note 10: The effect of the loading amount of SWCNTs on the proportion of the reversibly adsorbed, unadsorbed and irreversibly adsorbed SWCNTs in gel chromatography.

To investigate the effect of the loading amount of SWCNTs on the proportions of the reversibly adsorbed, unadsorbed and irreversibly adsorbed SWCNTs, different volumes of (6, 5) SWCNTs such as 1, 2, 5, 10, 15, 20 and 25 mL with a fixed concentration were loaded into gel columns of 40 mL equilibrated by 2 wt% SDS at 25 °C. The concentration of (6, 5) SWCNTs was characterized by optical absorption spectra (supplementary Fig. 24a). Then, the unadsorbed SWCNTs were eluted by co-surfactants of 0.5 wt% SDS/ 0.5 wt% SC solutions. The reversibly adsorbed SWCNTs were eluted by 5 wt% SDS solutions. In each case, the volumes of the unadsorbed and reversibly adsorbed SWCNTs were tuned into 40 mL and 30 mL, respectively. The corresponding optical absorption spectra are shown in supplementary Figs. 24b and c, respectively.

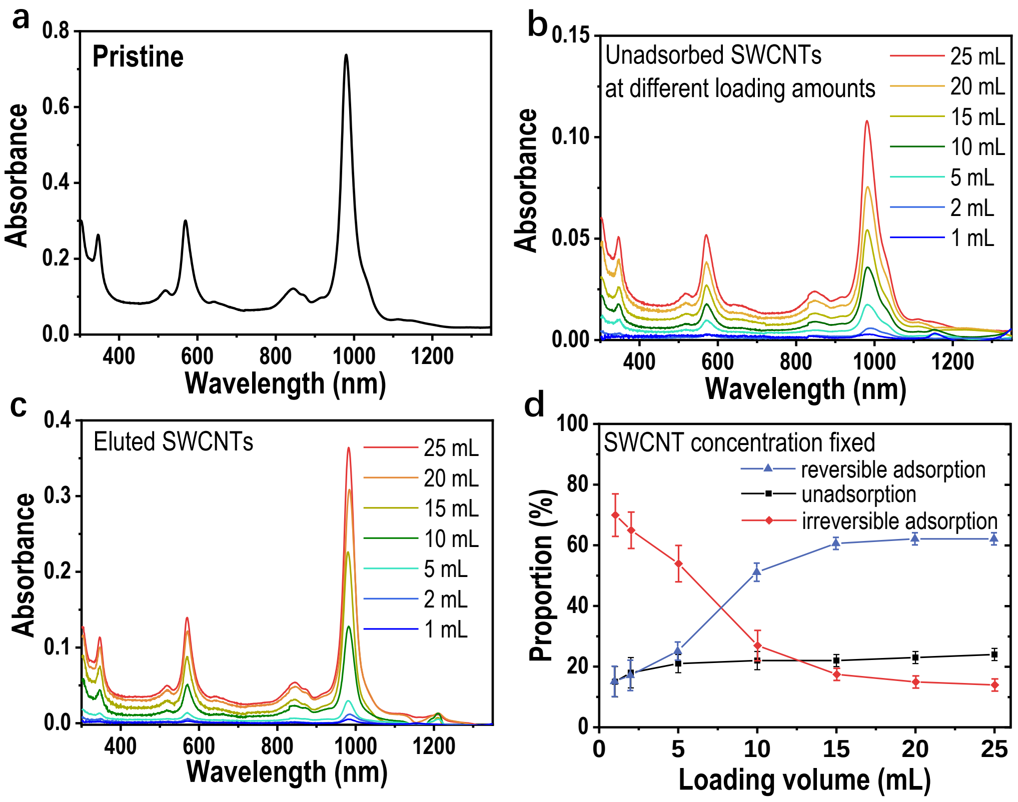

Supplementary Figure 24. The relationship between the reversibly adsorbed, unadsorbed and irreversibly adsorbed (6, 5) SWCNTs as a function of loading

amount. (a) The optical absorption spectrum of the separated (6, 5) SWCNTs as raw materials; Optical absorption spectra of (b) the unadsorbed SWCNTs and (c) eluted SWCNTs. The legend shows the volume of the loaded SWCNT solution. (d) The unadsorbed/irreversible adsorbed as a function of loading amount. Error bars in (d) are the standard deviation of statistics by repeating the experiment for three times.

The proportion change of the unadsorbed, reversibly adsorbed and irreversibly adsorbed parts as a function of the volumes of the loaded SWCNT solution was plotted in supplementary Fig. 24d. It is clear that the proportion of the unadsorbed SWCNTs increased from 15% to ~20% with increasing the loading amount from 1 to 5 mL, and subsequently increased slowly with a further increase in the loading amount. In contrast, the proportion of the irreversibly adsorbed fraction drops dramatically from 70% to ~20% with an increase in the loading amount from 1 to 15 mL and then decrease slowly when the loading amount further increases, which induced an increase in the proportion of the reversibly adsorbed SWCNTs.

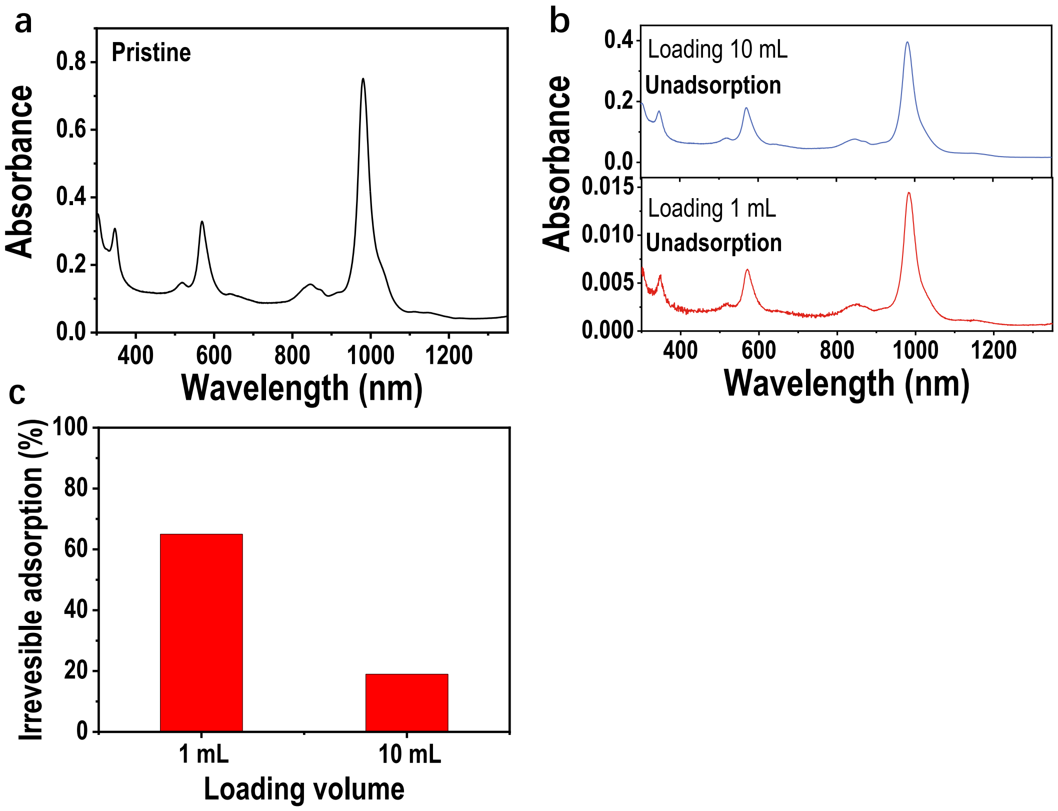

Supplementary Figure 25. Evaluation of the irreversible SWCNTs by loading (6,5) SWCNTs into different-volume gel column. Optical adsorption of 5 wt% SDS-(6, 5)

before a) and after b) loading to the gel columns. (c) Comparison of the proportion of irreversible adsorption at different loading amounts.

The high ratio of irreversible SWCNTs at a low loading amount indicates that the loaded SWCNTs preferentially adsorbed at irreversible adsorption sites possibly because of their stronger affinity. The irreversible adsorption may originate from a quasi-random binding event[14], which is highly related to the complex structure of gel being absent of region regularity in some regions[14-16]. As the loading amount increases, the proportion of irreversible part decreases possibly due to the occupation of irreversible sites. To further illustrate the origin of irreversible adsorption, we dispersed (6, 5) SWCNTs in 5 wt% SDS and loaded 1 mL and 10 mL of such solution to 40-mL gel columns equilibrated by 5 wt% SDS, respectively. The flow-through solution was collected as the unadsorbed part. The results are shown in supplementary Fig. 25. All the flow-through solutions were diluted to equal volume of 15 mL. Although the adsorption condition of semiconducting SWCNTs was not fulfilled at the concentration of 5 wt% SDS, irreversible adsorption was still observed, which may relate to the inhomongeneity of gel[14-16]. Similar to Eq. (1), irreversible binding process regardless of the environment condition can be described as follow:

$$SWCNT_{n,m}^{free} + \theta_{Ir} \xrightarrow{K_{n,m}^{Ir}} SWCNT_{n,m}^{Ir} \tag{4}$$

where θ_{Ir} and $K_{n,m}^{Ir}$ represent the number of binding sites of irreversible adsorption in the reaction volume and the forward rate constant of irreversible adsorption, respectively. Compared with reversible, irreversible adsorption sites exhibits stronger affinity with SWCNTs. When SWCNTs flow through gel column, they preferentially adsorb at the irreversible adsorption sites. During the elution process, Eq. (1) proceeds in the reverse direction, while Eq. (4) is still a forward reaction. Thus, SWCNTs eluted from reversible sites at the top of the gel column flow down and may be captured by irreversible sites at the bottom of the gel column. Conceptually, a gel column is usually considered a pile composed of many plates, each of which composes of a very thin gel layer[17]. Adsorption of SWCNTs in the upper plates leads to the occupation of both reversible and irreversible adsorption sites. Then, the remained SWCNTs in solution

flow down to the plates below. At low loading amounts, only plates at the top were filled. The solution that flows down contains much fewer SWCNTs, leaving the irreversible adsorption sites in the lower plates unoccupied. These unoccupied sites may trap SWCNTs that are eluted from the upper plates. As a result, nearly no SWCNTs were collected from the eluent. In contrast, when a large number of SWCNTs were loaded into the column, irreversible adsorption sites in the lower plates were filled. Thus, when the eluent flows through the column, the remained SWCNTs can be eluted from the lower plates. With an increase in loading amount, the proportion of irreversible adsorption decreases. This hypothesis coincides with the experimental results in supplementary Fig. 10, where the amount of SWCNTs separated from a gel recycled for 5 separation runs is higher than that of the new gel, because some of the irreversible adsorption sites have been occupied in the recycled gel.

Interestingly, the ratio of reversible and irreversible adsorption gradually reached a plateau with further increasing loading amount, as shown in supplementary Fig. 24d. As mentioned above, with increasing the loading amount of SWCNTs, the irreversible adsorption sites are occupied, resulting in a gradual decrease in the proportion of irreversible adsorption. However, increasing the loading amount also increase the possibility of irreversible adsorption of SWCNTs. For example, the impurities in SWCNT solutions such as amorphous carbon and bundles also increased with loading amount and form more irreversible adsorption[14]. The two aspects eventually hold the proportion of irreversible adsorption at ~ 15 %. Notably, this mechanism is established under the condition of normal loading. Overloading is supposed to be more complicated, involving the competition between different (*n, m*) species and the saturation of gel. Additionally, the form of irreversible adsorption may also be attributed to the inherent properties of some SWCNTs, such as length, defects and coatings[14].

In summary, increasing the concentration of raw SWCNT solution would increase the loading amount of each species, inevitably resulting in an increase in their separation yield.

Supplementary Note 11: Life cycle assessment (LCA) and techno-economic analysis (TEA) of single-chirality SWCNT production by separation.

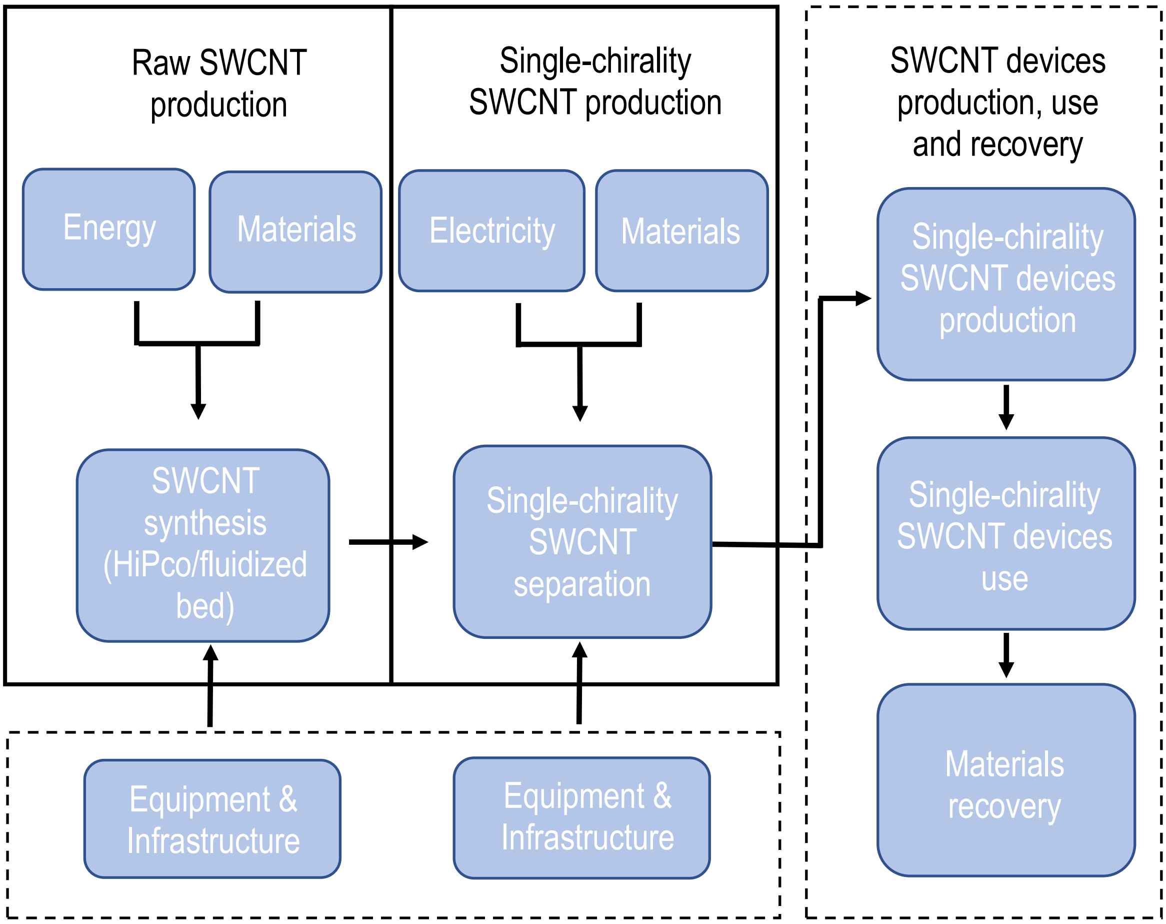

Supplementary Figure 26. Cradle-to-gate diagram of the LCA. Thick boxes show the system boundary of this LCA. Dotted boxes were not included in the current life cycle.

A LCA is carried out for environmental evaluation of separating single-chirality SWCNTs. The scope of this LCA is limited to the "cradle-to-gate", as indicated in supplementary Fig. 26, including the synthesis of raw SWCNTs and inputs of chemicals and energy for separation. Notably, the fabrication and application of SWCNT-based devices are excluded in life cycle stages because the environmental impacts and energy demand may significantly vary with different applications. The initial processes such as metal mining and, infrastructure building and equipment manufacturing were also excluded. To produce the life cycle inventory (LCI), materials and energy input to the separation process were measured from the experimental parameters, as shown in

supplementary Note 1. While, the LCI data involving commercial raw SWCNT production were referenced from the established literatures[18,19]. Life cycle impact assessment (LCIA) was performed on SimaPro 9.4. Ecoinvent 3.8 database and IPCC method were used for modeling LCIA[18-20].

11.1 Raw SWCNT production process

Under the assumption that carbon purity is identical, the greenhouse gas emissions and cumulative energy demand (CED) for synthesizing 1 g of SWCNTs were compared between different synthesis methods[18,19]. The results suggest that fluidized-bed chemical vapor deposition (CVD) has distinct advantages, as shown in supplementary Table 2. This is mainly due to the 3-diamentional (3D) growth in fluidized bed, which expands reaction space and increase the yield. In contrast, the synthesis of HiPco-SWCNTs generate large amount of CO_2 because of the reaction $2CO \leftrightarrow C + CO_2$. It is clear that raw SWCNTs prepared by fluidized-bed CVD is supposed to be more sustainable and greener, regardless of the results of separation[18].

Supplementary Table 2. Life cycle greenhouse gas emissions and cumulative energy demand for synthesizing 1 g of SWCNT by different methods

Synthesis methods	Greenhouse gas emissions (kg CO_2e./g)	Cumulative energy demand (CED, MJ)
**Fluidized-bed CVD[18]**	0.48	6.55
**HiPco-SWCNTs[18]**	33.87	442.12

11.2 Industrial separation process of single-chirality SWCNTs

As evidenced in the main text, the separation efficiency of single-chirality species is highly dependent on concentrations and structural distribution of raw SWCNTs. Due to the low concentration of SWCNT dispersion prepared by the traditional dispersion methods, it is difficult to achieve the mass separation of single-chirality species. To improve the separation efficiency and yield of single-chirality SWCNTs, we developed re-dispersion technique to prepare high-concentration SWCNT solutions. With this technique, the initial concentration of 4 mg/mL has been proved to be optimal concentration for the separation of single-chirality species from HiPco-SWCNTs. The raw G-SWCNTs synthesized by fluidized-bed CVD have wider structural distribution

and lower relative content of each (*n, m*) species than HiPco-SWCNTs, resulting that more energy and materials are required to separate 1 mg of specific single-chirality (*n, m*) SWCNTs. For comparison, we evaluated the inventory data of separating single-chirality species from HiPco- and G-SWCNTs with initial concentrations of 1 and 4 mg/mL, respectively. For the separation of HiPco-SWCNTs, 360 mL of SWCNT dispersion were loaded into columns filled with 900 mL (as described in supplementary Note 1.5). Similarly, 1600 mL of G-SWCNT solutions prepared with initial concentrations of 1 and 4 mg/mL applied into 900-mL gel columns. Because the yield of each (*n, m*) species is different, the average input and output to produce 1 mg of single-chirality SWCNTs were calculated by dividing material and energy consumptions by the total yield of the separated single-chirality SWCNTs (Supplementary Table 3). Notably, gel is usually recycled for 20 separation runs. Therefore, we assume that the consumption of gel is 1/20 of the gel volume employed per separation run. The SWCNTs precipitated by ultracentrifugation can be further dispersed for the separation of single-chirality species. The finally unadsorbed SWCNTs including metallic SWCNTs and large-diameter SWCNTs were recovered for further separation and application. Therefore, the precipitated SWCNTs by ultracentrifugation and unadsorbed SWCNTs are not included in the waste. The separation of SWCNTs was performed by an automatic system (AVANT 150, GE Healthcare). The separation temperature is controlled by a home-made thermostat.

Supplementary Table 3. Input and output of producing single-chirality species from HiPco-SWCNTs and G-SWCNT solutions with initial concentrations of 1 and 4 mg/mL.

	HiPco-SWCNTs		G-SWCNTs	
	1-mg/mL	4-mg/mL	1-mg/mL	4-mg/mL
Input[a]				
Volume of SWCNT solution (mL)	360	360	1600	1600
Mass of SWCNTs in solution (mg)	68.4	298.8	17.6	75.7
SDS (g)	805	805	842	842

SC (g)	419	419	297	297
DOC (g)	101	101	95	95
Deionized water (L)	133.8	133.8	123.5	123.5
Gel (mL)	900	900	900	900
Electricity (kW·h)	273	298	336	464
Time consumption [a](h)	135	151	184	264
Output[a]				
Waste surfactants in waste water(g)	883	883	920	920
Scrap Gel (mL)	225	225	225	225
Waste water containing surfactants (L)	93	93	95.4	95.4
Mass of the separated single-chirality species (mg)	2.86	12.5	0.18	2.47

Note: [a]Input, output and time consumption include the dispersion process, ultracentrifuge process and chromatography process.

We construct the LCA model on the assumption that surfactants input as chemicals organic, and the surfactant and scrap gel output as surfactant in water on the SimaPro platform. Waste water containing surfactant output as waste water. The LCI of commercial HiPco- and G-SWCNTs was reported in the previous work[18], as shown in supplementary Table 2. The greenhouse gas emissions and CED of producing 1-mg single-chirality SWCNTs are presented in Figure 6 in the main text.

11.3 Techno-economic analysis

We evaluated the financial life cycle costs based on materials, equipment and operating cost (Supplementary Tables 4-6) in China market. Since the current technique has not been industrialized and commercialized, we cannot define the market prices of the separated single-chirality SWCNTs. Therefore, the net present value is unavailable for the moment.

Supplementary Table 4. Material prices.

Materials	Quantity	Cost
HiPco-SWCNTs[a]	1 g	$550 USD
G-SWCNTs[b]	1 kg	$4200 USD

Water[c]	1 t	$1.26 USD
DOC[d]	1 kg	$1056.5 USD
SDS[d]	1 kg	$624.5 USD
SC[d]	1 kg	$1275.4 USD
Electricity[e]	1 kW·h	$0.13 USD
Gel[f]	750 mL	$1003.2 USD

Note: [a]HiPco was purchased from NanoIntegris. [b]G-SWCNT was provided by Beijing North Guonemg Technology Co., Ltd.(http://www.gnnano.com) [c]The water price of industry and commerce of Beijing in 2022. [d]From Sigma-Aldrich. [e]The single price of general industry and commerce of Beijing Economic-Technological Development Area in 2022. [f]From GE Healthcare

Supplementary Table 5. Cost for equipment.

Equipment	Reference capacity	Reference cost[a]
**Homogenizer, Branson 450D[b]**	Power 400 W	$59124 USD
**Ultracentrifuge[c]**	210,000 g 120 mL	$36231 USD
**Water purification system[d]**	180 L per day	$14492 USD
**Chromatography system[e]**	Coupling with 5 columns	$168920 USD
**Column[e]**	1170 mL	$1859 USD

Note: [a]The reference cost is evaluated according to the prices when we purchase the corresponding equipment. [b]Tip-type homogenizer was purchased from Branson, and was equipped with a thermostat. Four sets of homogenizers were employed for mass dispersion and separation. [c]From Hitachi. [d]From Merck Milli-Q. [e]The chromatography system includes a set of High-Performance Liquid Chromatography and a thermostat. The High-Performance Liquid Chromatography and columns were purchased from GE Healthcare. The thermostat was purchased from Giant Force Instrument Enterprise Co., Ltd.

Supplementary Table 6. Operating cost

Elements	Cost
**Maintenance[a]**	
**Homogenizer, Branson 450D**	$0.6 USD per hour
**Ultracentrifuge**	$0.3 USD per hour
**Water purification system**	$0.3 USD per hour
**Chromatography system**	$0.6 USD per hour

Column	/
**Depreciation**[b]	
**Homogenizer, Branson 450D**	$0.9 USD per hour
**Ultracentrifuge**	$0.5 USD per hour
**Water purification system**	$0.2 USD per hour
**Chromatography system**	$2.5 USD per hour
**Column**	$0.03 USD per hour
**Operating materials**[c]	
**Replaceable ultrasonic homogenizer tips**	$0.7 USD per hour
**Centrifuge tubes**	$0.08 USD per hour
**Filter of pure water system**	$0.2 USD per hour
**Labor costs**[d]	$10 per hour

Note: [a]The cost of maintenance of equipment is empirically estimated under the condition that each equipment runs for 90 h a week. [b]We assume that the annual depreciation of all equipment is 7%[7], under the condition that each equipment runs for 90 h a week. Thus, the average depreciation of each equipment per hour is calculated. [c]The operating materials are purchased from Hitachi, Branson and Merck Milli-Q, respectively. The costs of operating materials are estimated by dividing the price by the operation lifetime of each operating material. [d]Labor cost was estimated at ~$10 per hour based on the average manufacturing salary in Beijing in 2021.

The deionized water is produced by the water purification system. Tap water was used as the source of water and the output pure water is one third. Based on the supplementary Tables 3-4, the costs of various materials for producing 1-mg single-chirality species were calculated, as shown in supplementary Fig. 27. It can be seen that, with an increase in the initial concentration of SWCNTs to 4 mg/mL, the costs of material and energy including surfactants, gel and electricity of producing 1-mg single-chirality species decrease rapidly. When high-concentration HiPco-SWCNTs were used as raw materials, the raw SWCNT cost of producing 1-mg single-chirality species is $13.2, and accounts for about 2.5% of all material costs. In contrast, the cost on G-SWCNTs is only $0.13, which is only 1% of that of HiPco-SWCNTs to produce 1-mg single-chirality SWCNTs.

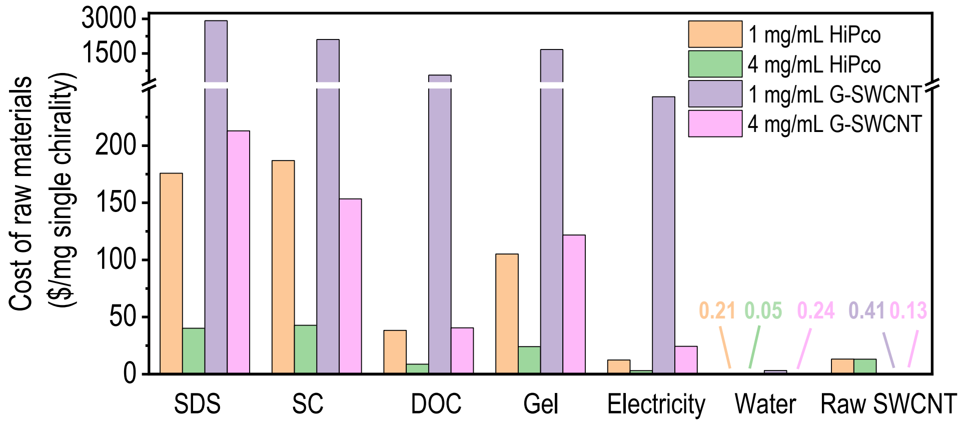

Supplementary Figure 27. Costs of various raw materials of separating 1-mg single-chirality SWCNTs from HiPco- and G-SWCNTs.

Basing on supplementary Table 6, the operating cost was evaluated by multiplying the operating time of each equipment and the corresponding cost on maintenance, depreciation and operating materials. Specifically, $Cost_o = 2.2 \times t_1 + 0.88 \times t_2 + 3.83 \times t_3 + 10 \times t_4$, where t_1, t_2, t_3 and t_4 represent the operating time of sonication system, ultracentrifuge, separation system (composed of chromatography system, column and water purification system) and working hours of employees. The corresponding operating time is determined by experimental parameters in supplementary Note 1 and Table 3.

In the absence of reference, inflation, interest of loan, tax credits and infrastructure building are excluded. Thus, the total cost of 1-mg single-chirality is defined as: $Cost_{total} = (Cost_m + Cost_o)/yield$, where the $Cost_m$ and $Cost_o$ are the total cost on raw materials, and operating costs. The resulted costs of producing 1-mg single-chirality SWCNTs for different raw materials and different concentrations are presented in Fig. 6c.

We can imagine that the cost of producing single-chirality species would further reduce with scaling up. For instance, when the equipment with 10 times capacity including sonication dispersion, ultracentrifugation and separation systems are employed, and 80 g of G-SWCNTs or 18 g of HiPco-SWCNTs are input in the

separation, the separation yield must be multiplied. In other words, the time required to produce 1-mg single-chirality SWCNTs must be greatly reduced. At the same time, the cost of materials and energy consumed per 1 mg of single-chirality SWCNTs, as well as waste output and labor costs, will be significantly reduced. A benchmark identifying the features of each method is quite necessary for industrial separation. However, other reported methods, such as such as ultracentrifugation and aqueous two-phase extraction ATPE, have not disclosed their yield and productivity[21,22]. Therefore, it cannot be systematically compared with other separation methods at present.

Supplementary References:

1. S. Halelfadl, P. Estellé, B. Aladag, N. Doner, T. Maré, Viscosity of carbon nanotubes water-based nanofluids: Influence of concentration and temperature. Int. J. Therm. Sci, 7, 111-117 (2013).
2. G. Wernet, C. Bauer, B. Steubing, J. Reinhard, E. Moreno-Ruiz, B. Weidema, The ecoinvent database version 3 (part I): overview and methodology. *Int. J. Life Cycle Assess.* **21,** 1218−1230 (2016).
3. Vakarelski, I. U. Brown, S. C. Rabinovich, Y. I. & Moudgil, B. M. Lateral force microscopy investigation of surfactant-mediated lubrication from aqueous solution. *Langmuir* **20,** 1724-1731 (2004).
4. Li, J. *et al.* AFM studies on liquid superlubricity between silica surfaces achieved with surfactant micelles. *Langmuir* **32,** 5593−5599 (2016).
5. Liu, H., Nishide, D., Tanaka, T. & Kataura, H. Large-scale single-chirality separation of single-wall carbon nanotubes by simple gel chromatography. *Nat. Commun.* **2,** 309 (2011).
6. Wei, X. et al. Experimental determination of excitonic band structures of single-walled carbon nanotubes using circular dichroism spectra. *Nat. Common.* **7,** 12899 (2016).
7. Liu, H., Tanaka, T., Urabe, Y. & Kataura, H. High-efficiency single-chirality separation of carbon nanotubes using temperature-controlled gel chromatography.

Nano Lett. **13,** 1996-2003 (2013).

8. Yang, D. et al. Submilligram-scale separation of near-zigzag single-chirality carbon nanotubes by temperature controlling a binary surfactant system. *Sci. Adv.* **7,** eabe0084 (2021).
9. Yang, D. et al. Structure sorting of large-diameter carbon nanotubes by NaOH tuning the interactions between nanotubes and gel. *Adv. Funct. Mater.* **27,** 1700278 (2017).
10. Helfferich, F. G. & Hwang, Y.-L. Ion exchange kinetics, In *Ion Exchangers* (De Gruyter, Berlin, 1991).
11. Tvrdy, K. et al. A kinetic model for the deterministic prediction of gel-based single-chirality single-walled carbon nanotube separation. *ACS Nano* **7,** 1779-1789 (2013).
12. Rebecca K. L. & Giorgio C. Protein transport in constrained anionic hydrogels: diffusion and boundary-layer mass transfer. *Ind. Eng. Chem. Res.* **40,** 1548-1558 (2001).
13. Shastry, T. A. Morris-Cohen, A. J. Weiss, E. A. & Hersam, M. C. Probing carbon nanotube−surfactant interactions with two-dimensional DOSY NMR. *J. Am. Chem. Soc.* **135,** 6750−6753 (2013).
14. Watts, B. P, Rolsma, C., Dolan, M. & Tvrdy, K. Mechanism and mitigation of irreversible material loss within gel-based single-walled carbon nanotube purification schemes. *J. Phys. Chem. C* **125,** 26084-26098(2021).
15. E. Antoniou, M. Tsianou, Solution properties of dextran in water and in formamide. *J. Appl. Polym. Sci.* **125,** 1681−1692 (2012).
16. K. A. Granath, Solution properties of branched dextrans. *J. Colloid Sci.* **13,** 308−328 (1958).
17. Glueckauf, E. Theory of chromatography. Part 9. The "theoretical plate" concept in column separations. *Trans. Faraday Soc.* **51**, 34-44 (1955).
18. Teah, H. Y. *et al.* Life cycle greenhouse gas emissions of long and pure Carbon nanotubes synthesized via on-substrate and fluidized-bed chemical vapor deposition. *ACS Sustainable Chem. Eng.* **8,** 1730−1740 (2020).

19. Healy, M. L., Dahlben, L. J. & Isaacs, J. A. Environmental assessment of single-walled carbon nanotube processes. *J. Ind. Ecol.* **12,** 376−393 (2008).

20. Gavankar, S., Suh, S. & Keller, A. A. The Role of Scale and Technology Maturity in Life Cycle Assessment of Emerging Technologies: A Case Study on Carbon Nanotubes. *J. Ind. Ecol.* **19,** 51−60 (2015).

21. S. Ghosh, S. M. Bachilo, R. B. Weisman, Advanced sorting of single-walled carbon nanotubes by nonlinear density-gradient ultracentrifugation. *Nat. Nanotechnol.* **5,** 443-450 (2010).

22. G. Ao, J. Streit, J. Fagan, M. Zheng, Differentiating left- and right-handed carbon nanotubes by DNA. *J. Am. Chem. Soc.* **138,** 16677-16685 (2016).
