# Supplementary figures and images for: Preparing high-concentration individualized carbon nanotubes for industrial separation of multiple single-chirality species
Source: Nat Commun. 2023 Apr 29;14:2491. doi: 10.1038/s41467-023-38133-0 (PMC10148823; doi:10.1038/s41467-023-38133-0)

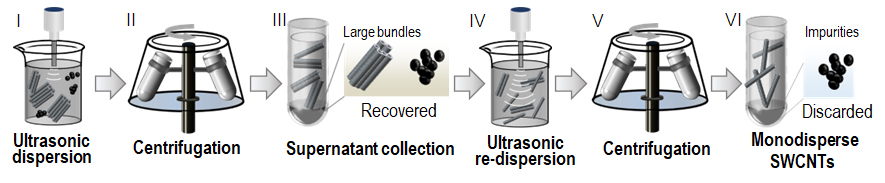

Supplement: Supplementary file 3 — Source Data [file 41467_2023_38133_MOESM3_ESM.zip › 7 Source data/Figure 1/1a/Figure 1a-disperdion provess of SWCNT solution.jpg]

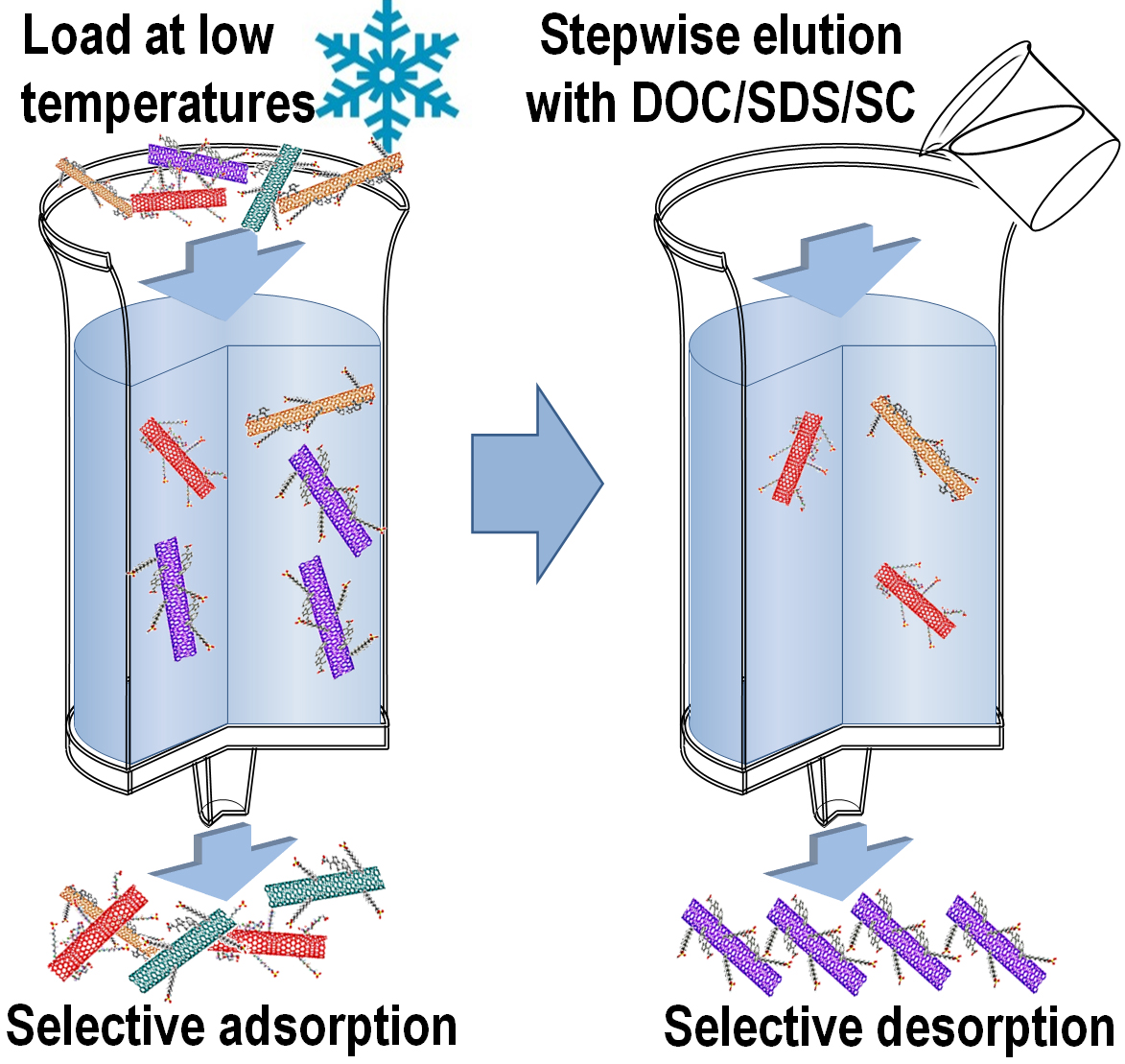

Supplement: Supplementary file 3 — Source Data [file 41467_2023_38133_MOESM3_ESM.zip › 7 Source data/Figure 2/Figure 2a/Figure 2a-separation process.jpg]

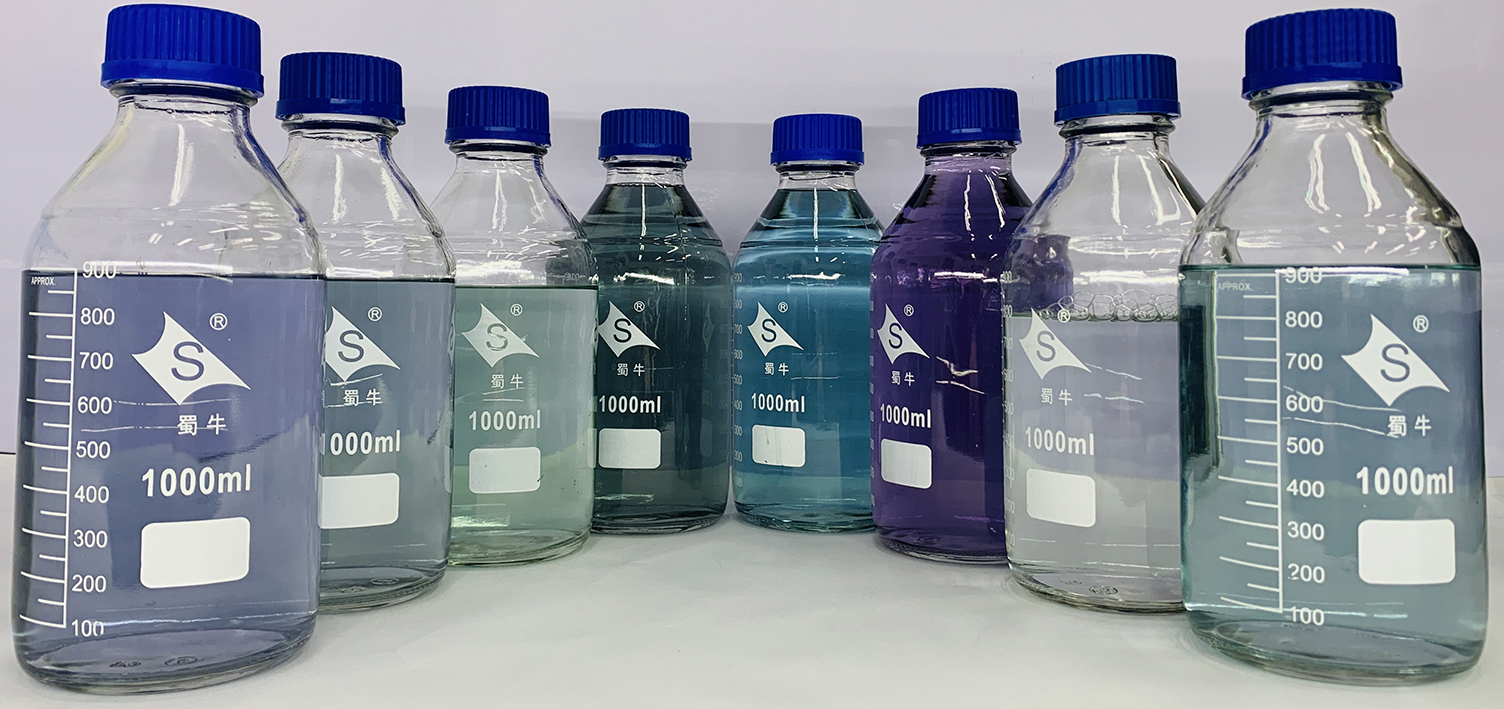

Supplement: Supplementary file 3 — Source Data [file 41467_2023_38133_MOESM3_ESM.zip › 7 Source data/Figure 2/Figure 2e/Solution photos of single-chirality species7.jpg]

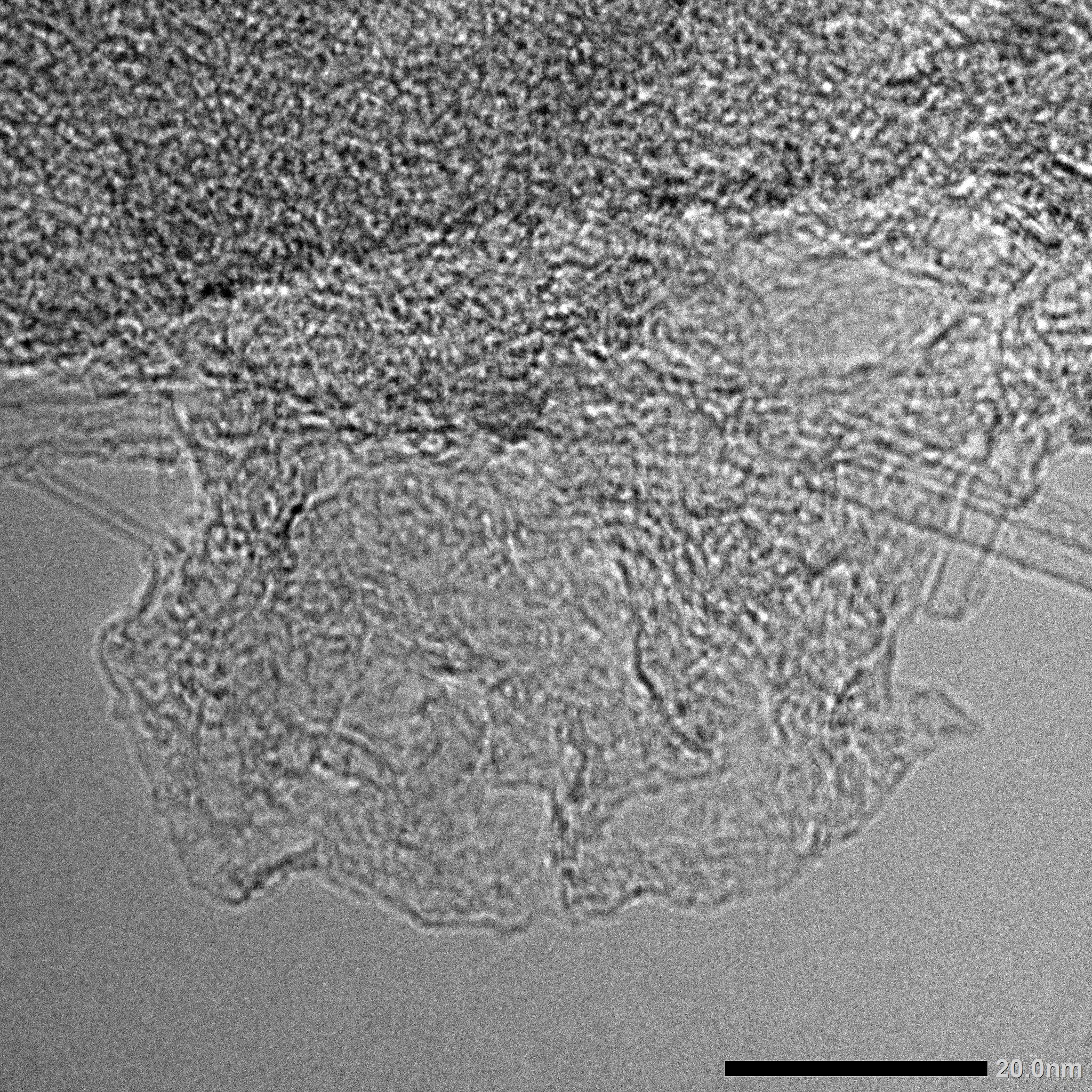

Supplement: Supplementary file 3 — Source Data [file 41467_2023_38133_MOESM3_ESM.zip › 7 Source data/Figure 3/Figure 3c-TEM of G-SWCNTs.jpg]

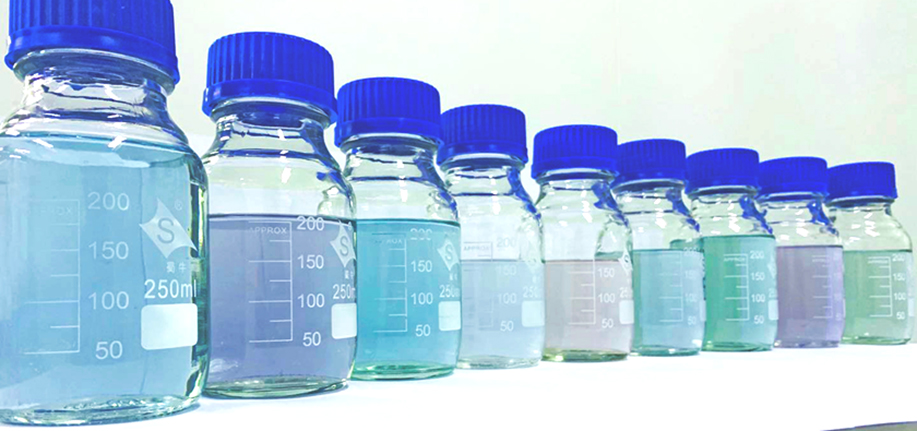

Supplement: Supplementary file 3 — Source Data [file 41467_2023_38133_MOESM3_ESM.zip › 7 Source data/Figure 4/Figure 4c-Solution photo.jpg]

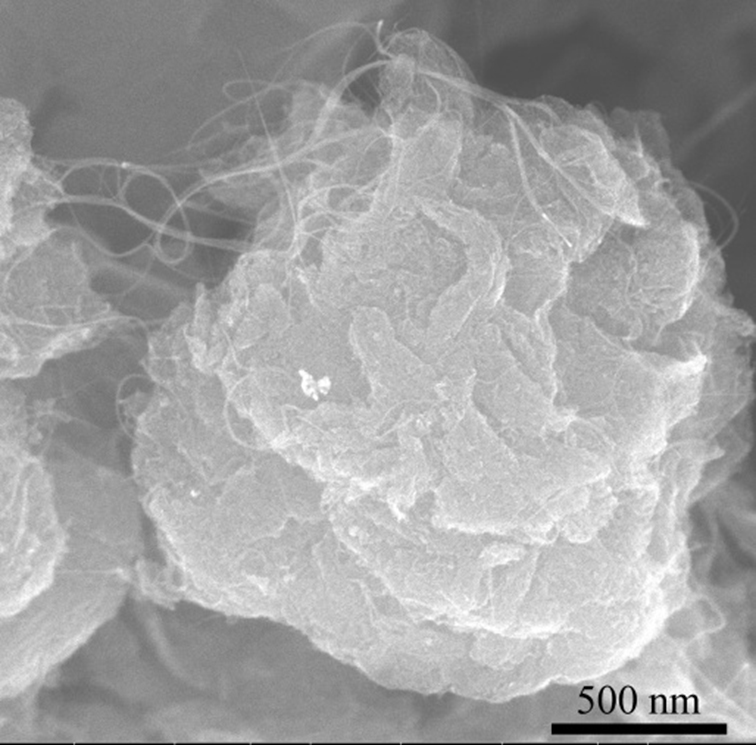

Supplement: Supplementary file 3 — Source Data [file 41467_2023_38133_MOESM3_ESM.zip › 7 Source data/Supplementary Figure 11/Figure 11a.jpg]

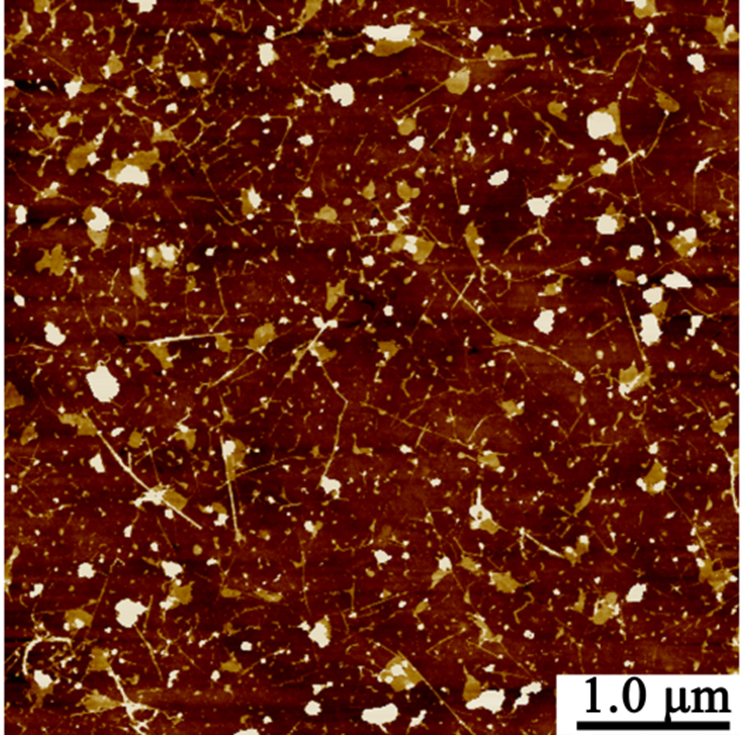

Supplement: Supplementary file 3 — Source Data [file 41467_2023_38133_MOESM3_ESM.zip › 7 Source data/Supplementary Figure 11/Figure 11b.jpg]

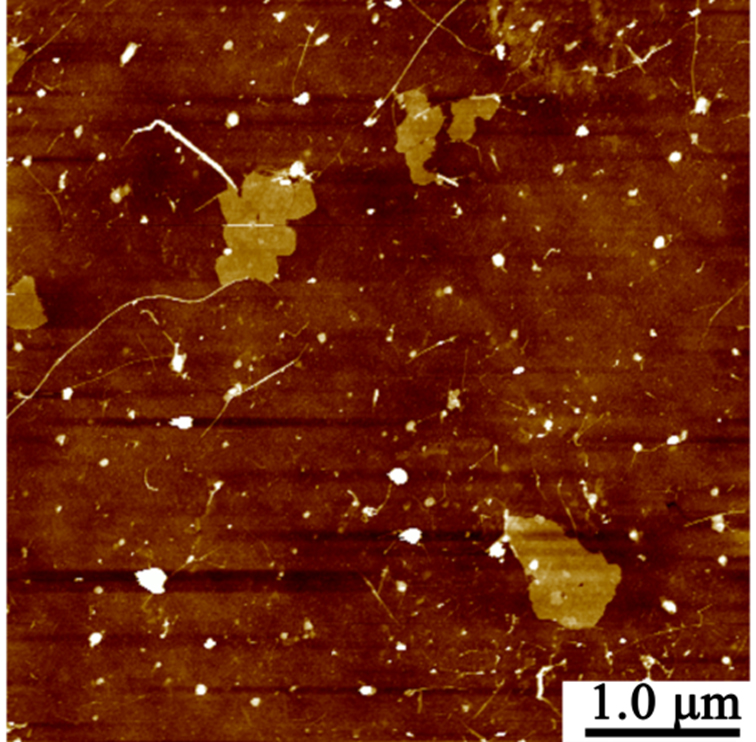

Supplement: Supplementary file 3 — Source Data [file 41467_2023_38133_MOESM3_ESM.zip › 7 Source data/Supplementary Figure 11/Figure 11c.jpg]

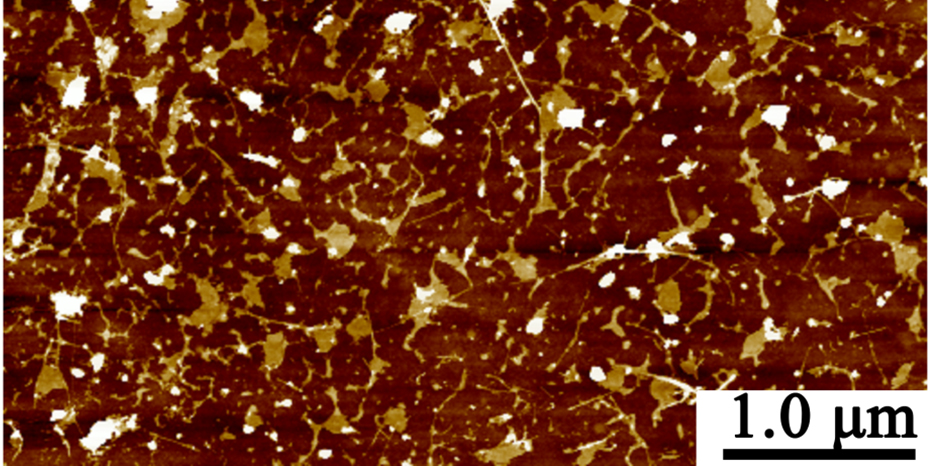

Supplement: Supplementary file 3 — Source Data [file 41467_2023_38133_MOESM3_ESM.zip › 7 Source data/Supplementary Figure 17/Supplementary Figure 17a-AFM of unadsorbed SWCNTs.jpg]

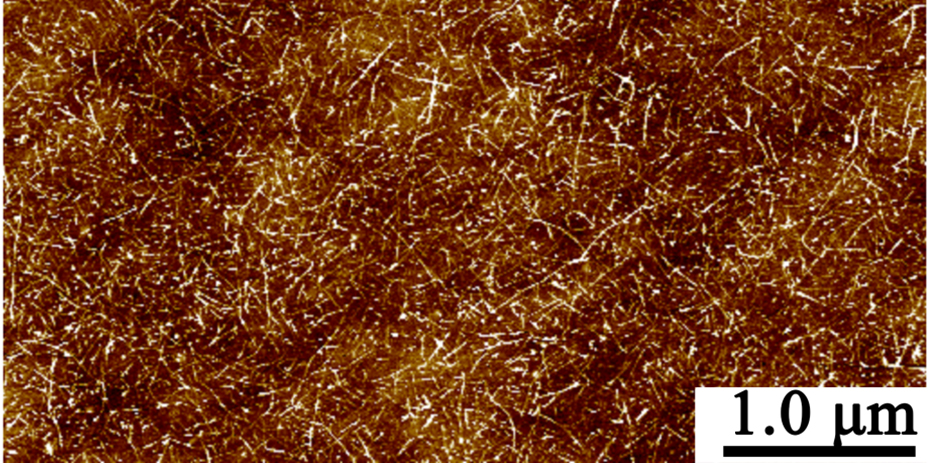

Supplement: Supplementary file 3 — Source Data [file 41467_2023_38133_MOESM3_ESM.zip › 7 Source data/Supplementary Figure 17/Supplementary Figure 17b-AFM of adsorbed SWCNTs.jpg]

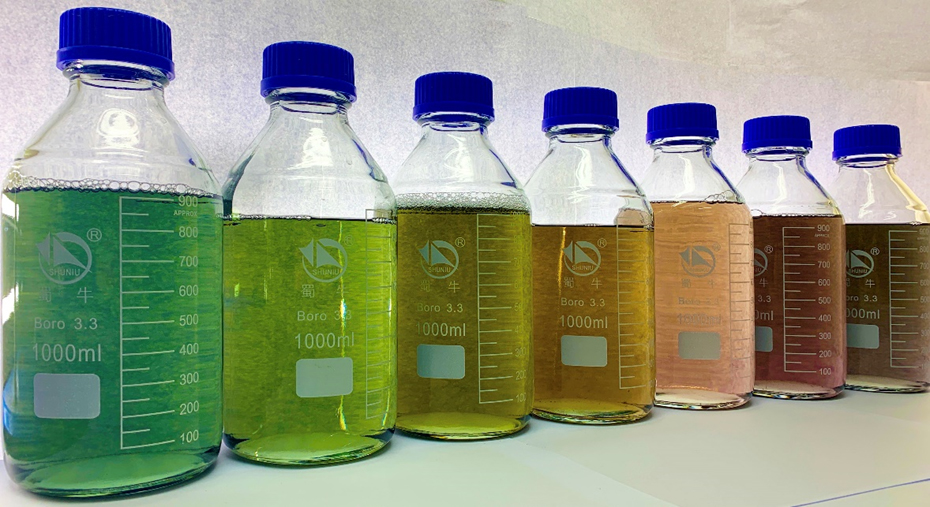

Supplement: Supplementary file 3 — Source Data [file 41467_2023_38133_MOESM3_ESM.zip › 7 Source data/Supplementary Figure 18/Supplementary Figure 18b-Solution photo of different diameter S-SWCNTs.jpg]

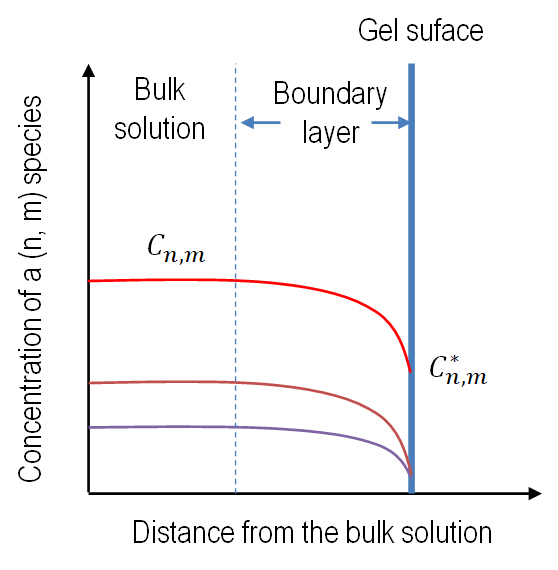

Supplement: Supplementary file 3 — Source Data [file 41467_2023_38133_MOESM3_ESM.zip › 7 Source data/Supplementary Figure 22/Supplementary Figure 22-mass transfer of SWCNTs.jpg]

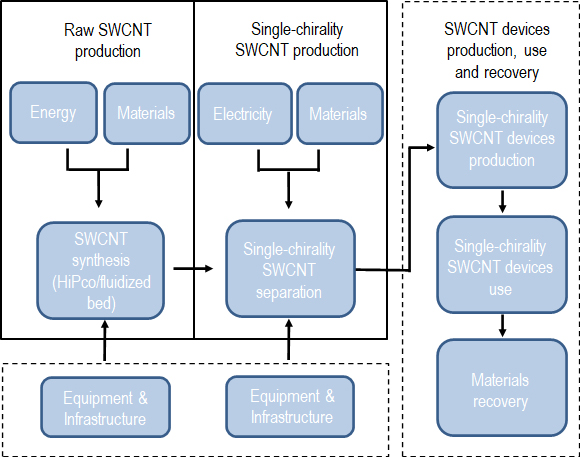

Supplement: Supplementary file 3 — Source Data [file 41467_2023_38133_MOESM3_ESM.zip › 7 Source data/Supplementary Figure 26/Supplementary Figure 26-Cradle-to-gate diagram of the LCA.jpg]

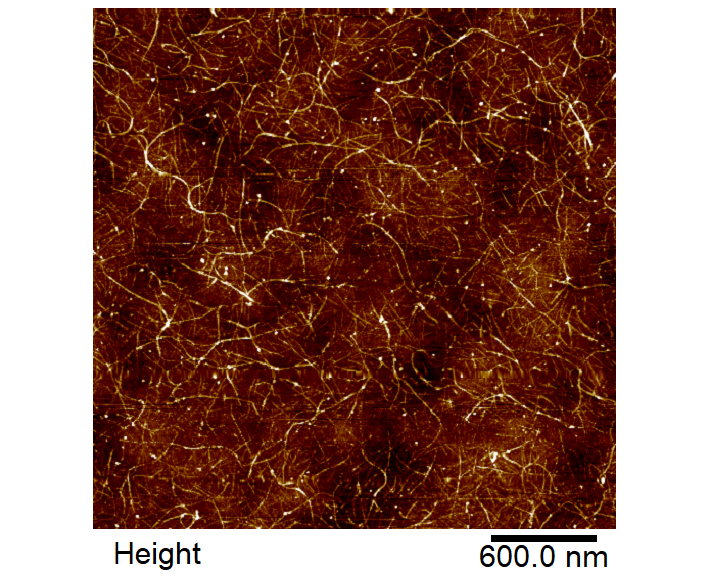

Supplement: Supplementary file 3 — Source Data [file 41467_2023_38133_MOESM3_ESM.zip › 7 Source data/Supplementary Figure 3/Figure 3d-AFM of SWCNTs prepared by traditional method.tif]

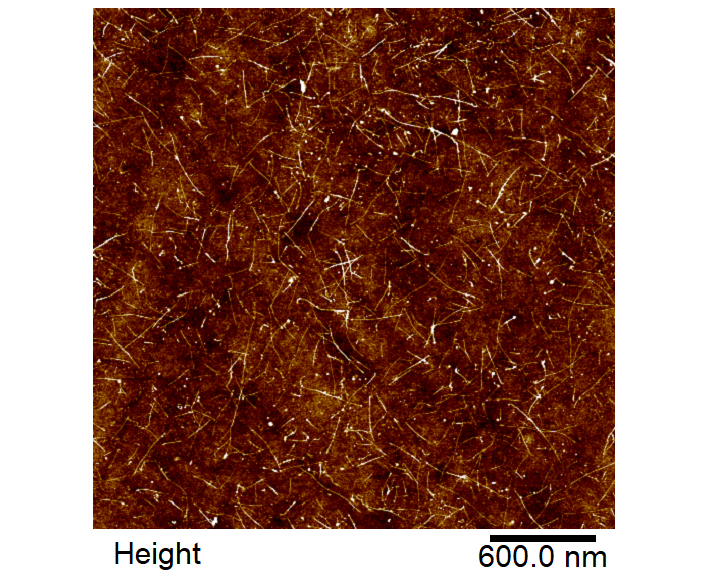

Supplement: Supplementary file 3 — Source Data [file 41467_2023_38133_MOESM3_ESM.zip › 7 Source data/Supplementary Figure 3/Figure 3e-AFM of SWCNTs prepared by re-dispersion.tif]

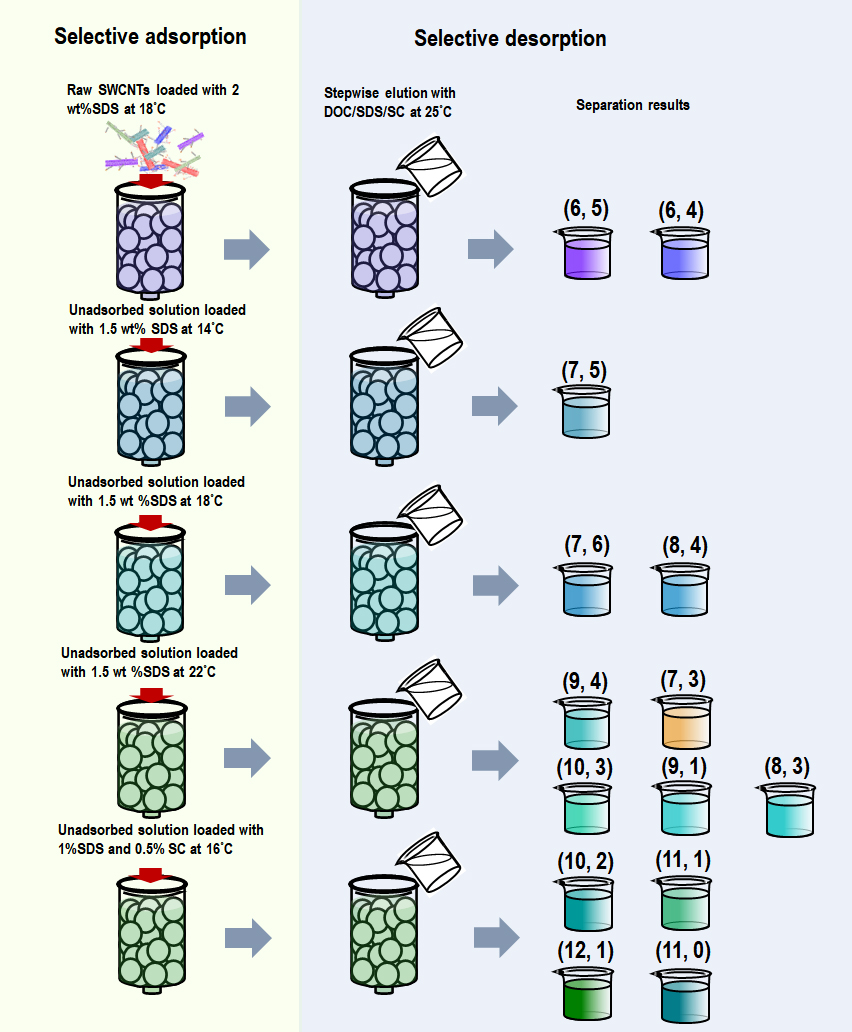

Supplement: Supplementary file 3 — Source Data [file 41467_2023_38133_MOESM3_ESM.zip › 7 Source data/Supplementary Figure 4/Supplementary Figure 4-Schematic diagram of separation process.jpg]

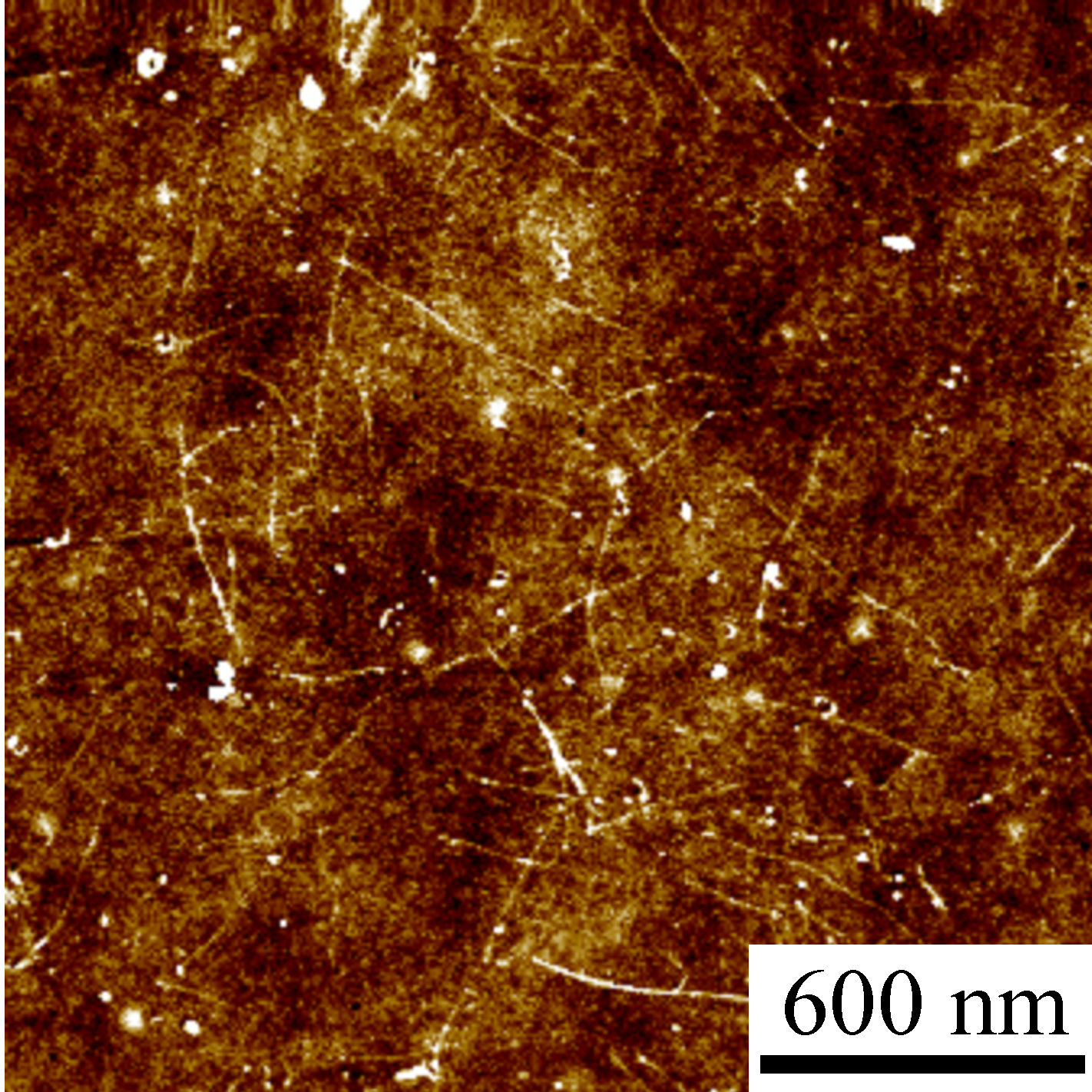

Supplement: Supplementary file 3 — Source Data [file 41467_2023_38133_MOESM3_ESM.zip › 7 Source data/Supplementary Figure 9/Figure 9a-AFM of SWCNTs dispersed for 9 hours.tif]

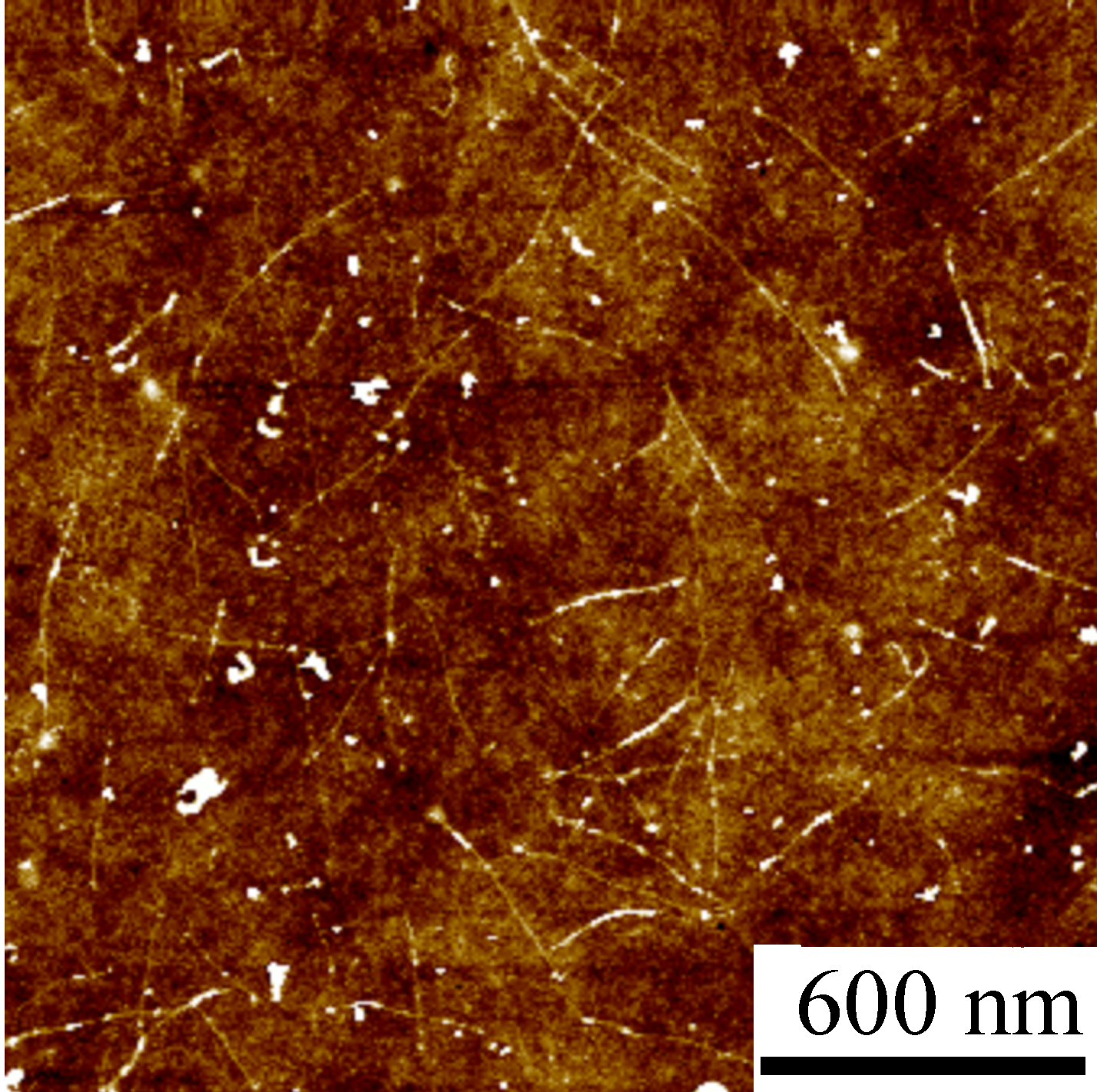

Supplement: Supplementary file 3 — Source Data [file 41467_2023_38133_MOESM3_ESM.zip › 7 Source data/Supplementary Figure 9/Figure 9c-AFM of SWCNTs dispersed for 24 hours.tif]
